# Supplementation of live yeast based feed additive in early life promotes rumen microbial colonization and fibrolytic potential in lambs

**DOI:** 10.1038/s41598-019-55825-0

**Published:** 2019-12-16

**Authors:** Frédérique Chaucheyras-Durand, Aurélie Ameilbonne, Pauline Auffret, Mickaël Bernard, Marie-Madeleine Mialon, Lysiane Dunière, Evelyne Forano

**Affiliations:** 1 Lallemand SAS, 31702 Blagnac, France; 2 Université Clermont Auvergne, INRA, UMR 454 MEDIS, F-63000 Clermont-Ferrand, France; 3 UE 1414 Herbipôle, INRA Auvergne Rhône Alpes, F-63122 Saint-Genès Champanelle, France; 4 Université Clermont Auvergne, INRA, VetAgro Sup, UMR 1213 Herbivores, F-63000 Clermont-Ferrand, France; 5 Present Address: Ifremer, UMR, 241 EIO Tahiti, French Polynesia

**Keywords:** Microbial ecology, Microbial communities

## Abstract

Rumen microbiota is of paramount importance for ruminant digestion efficiency as the microbial fermentations supply the host animal with essential sources of energy and nitrogen. Early separation of newborns from the dam and distribution of artificial milk (Artificial Milking System or AMS) could impair rumen microbial colonization, which would not only affect rumen function but also have possible negative effects on hindgut homeostasis, and impact animal health and performance. In this study, we monitored microbial communities in the rumen and the feces of 16 lambs separated from their dams from 12 h of age and artificially fed with milk replacer and starter feed from d8, in absence or presence of a combination of the live yeast *Saccharomyces cerevisiae* CNCM I-1077 and selected yeast metabolites. Microbial groups and targeted bacterial species were quantified by qPCR and microbial diversity and composition were assessed by 16S rDNA amplicon sequencing in samples collected from birth to 2 months of age. The fibrolytic potential of the rumen microbiota was analyzed with a DNA microarray targeting genes coding for 8 glycoside hydrolase (GH) families. In Control lambs, poor establishment of fibrolytic communities was observed. Microbial composition shifted as the lambs aged. The live yeast supplement induced significant changes in relative abundances of a few bacterial OTUs across time in the rumen samples, among which some involved in crucial rumen function, and favored establishment of *Trichostomatia* and *Neocallimastigaceae* eukaryotic families. The supplemented lambs also harbored greater abundances in *Fibrobacter succinogenes* after weaning. Microarray data indicated that key cellulase and hemicellulase encoding-genes were present from early age in the rumen and that in the Supplemented lambs, a greater proportion of hemicellulase genes was present. Moreover, a higher proportion of GH genes from ciliate protozoa and fungi was found in the rumen of those animals. This yeast combination improved microbial colonization in the maturing rumen, with a potentially more specialized ecosystem towards efficient fiber degradation, which suggests a possible positive impact on lamb gut development and digestive efficiency.

## Introduction

The microbiota of the gastrointestinal tract (GIT) is of primary importance in nutrition and health of all mammals including ruminants. In ruminants, microbial inoculation of the rumen starts immediately at birth, from the vaginal canal, udder skin, colostrum^1^ but also fecal material and saliva from the dam^2^. Members of typical functional ruminal populations, such as methanogens or fibrolytic bacteria, have been detected in the rumen of calves less than 20 minutes after birth^3^ and are metabolically active^4^. More than 8,000 proteins produced by the rumen microbiota have actually been found in pre-weaned calves^5^. Rapid changes occur in the composition of the ruminal bacterial community during the first days of life^6,7^. In the hindgut of calves, Bacteroidetes, Firmicutes and Proteobacteria are detected at birth, and the microbial pattern evolves with age and with intake of starter feed^8^. GIT microbiota plays important roles in gastrointestinal development and function, immune system maturation, and pathogen resistance mechanisms^9^.

In current commercial dairy systems, early maternal separation (within a few hours) of the newborn combined with distribution of milk replacer (MR), which could be defined as Artificial Milking System (AMS) are put in place in order to maximize farmer revenue from raw milk. Moreover, in ovine breeding systems, highly prolific ewes very commonly give birth to 3 to 4 lambs. Generally the less vigorous or smaller ones are directed to AMS. These rearing practices induce animal stress and increase the risk that young ruminants suffer from digestive problems during the pre-weaning phase, which may affect growth performance, and deteriorate gut health with pathogen emergence. These troubles might be linked to perturbations of the maturation of the digestive tract, correlated with poor rumen/intestine colonization by functional beneficial microbial communities after birth. Indeed, deficiencies in sustainable establishment of key members of the rumen microbiota have been reported in AMS with subsequent impaired growth capacities of lambs^10–12^. Moreover, AMS has been reported to have a negative effect on sanitary score^13^ which may be due to microbial dysbiosis in the lower gut segments. In this context, strategies aiming at promoting optimal microbial colonization of the gastrointestinal tract could be of interest.

In ruminants, research studies focusing on the developing rumen have demonstrated that it may be possible to regulate the microbial community development by controlling the feeding management early in life, with subsequent effects later in time^7,14,15^. Previous research conducted in our team showed that the distribution of a live yeast additive very early in age could accelerate microbial establishment of functional communities in the rumen of lambs reared with their dams^16^ and stimulate the set-up of fibrolytic bacteria in gnotobiotically-reared lambs maintained into sterile isolators^17^. However, the effect of this yeast additive has not been studied in the context of AMS, yet. Therefore, we postulated that the distribution of live yeast additive early in life in lambs separated from their dams and fed with MR would positively influence microbial establishment in the gastro-intestinal tract. The purpose of this study was thus to monitor microbial communities in the gastro-intestinal tract of lambs fed with MR during the two first months of age, in absence or presence of live yeast additives in their diet. We measured the development of microbial diversity, abundance, composition of Bacteria, Archaea and Eukaryotes, and of the fibrolytic potential of the microbiota in the rumen as a proxy of rumen function and digestive capacity and in the feces as a proxy of gut health.

## Results

### Control of yeast populations in the experimental pellets

Viable yeast counts were quantified in the experimental pellets. The measured concentrations were in line with expectations (expected concentration was ~6.8 Log_10_ CFU/g of feed), taking into account a 0.5 Log_10_ tolerance margin which is generally accepted for microbial additives^18^. There was no decrease in yeast concentration over time, demonstrating the stability of the supplemented feed. In the non-supplemented feed, wild yeasts were detected at a concentration below 3.0 Log_10_ CFU/g of feed.

### Lamb growth

Two lambs died in each group during the experiment: two lambs (one from each group) died before 10 days of age, probably because they were not able to adapt to AMS. One died at 6 weeks from a not enteric infectious problem (Control group), one died at 3 weeks from sudden death, which could not be attributed to any digestive or respiratory disease (Supplemented group). Therefore, growth curves and average daily gain (ADG) calculations were done including the 6 remaining lambs per group. Otherwise, lambs were in a good sanitary status throughout the study. Individual weights were slightly more heterogeneous in the Supplemented group as shown by higher standard deviations (Fig. S1 and Table 1), however, no significant difference was observed between groups in body weight, and in ADG (Table 1).

**Table 1:** Growth parameters and rumen VFA concentrations and proportions for both groups.

Measured parameters		Control	Supplemented
*Growth*			
Birth weight (kg)		3.52 ± 0.09	3.40 ± 0.13
BW (kg) d 10		4.42 ± 0.23	4.60 ± 0.88
d 20		7.42 ± 0.59	7.12 ± 1.31
d 40		15.36 ± 1.79	14.01 ± 2.16
d 60		18.50 ± 2.03	17.13 ± 3.16
ADG (kg/d) 0–60 d		0.25 ± 0.03	0.23 ± 0.05
*Rumen fermentative parameters*			
Total VFA (mM)	d 5	33.76 ± 12.32	34.43 ± 14.15
	d 10	44.66 ± 11.52	36.97 ± 8.63
	d 42	56.84 ± 18.92	61.62 ± 6.93
	d 56	47.00 ± 8.67	68.23 ± 25.81
Acetate (%)	d 5	71.52 ± 3.20	68.50 ± 7.20
	d 10	72.69 ± 4.97	61.60 ± 4.80
	d 42	68.74 ± 5.57	71.98 ± 5.45
	d 56	70.01 ± 5.17	68.81 ± 4.06
Propionate (%)	d 5	20.42 ± 3.85	24.25 ± 6.89
	d 10	18.27 ± 1.95	25.59 ± 3.15
	d 42	16.78 ± 2.93	15.45 ± 3.10
	d 56	13.89 ± 2.50	19.90 ± 5.26
Butyrate (%)	d 5	4.11 ± 1.23	4.00 ± 3.70
	d 10	4.78 ± 2.04	6.20 ± 1.91
	d 42	7.01 ± 0.99	5.81 ± 1.63
	d 56	8.31 ± 2.95	5.36 ± 1.80

### Rumen fermentative parameters

Large individual variations were observed for ruminal pH from birth (Fig. S1). The mean pH decreased with time until day 28 in both groups then it increased again up to 60 days of age. Due to large individual variability, no difference in mean pH values was highlighted between groups.

The average VFA concentration (Table 1) was numerically higher in the rumen of Supplemented lambs compared to Controls at d56 (P = 0.16). The VFA mixture was composed by acetate, propionate and butyrate with variations according to individual, age and group (Table 1). The acetate proportion was fairly stable with age, the propionate percentage decreased (P < 0.01) but remained higher in the Supplemented group (P < 0.01), and the butyrate proportion tended to increase with age (P = 0.056).

### Characteristics of the lamb microbiota across time in the rumen and the feces

#### Quantification of prokaryotes by qPCR

Bacteria were detected in the rumen of all lambs from 2 days of age at high population levels, between 8 and 9 Log_10_ copies of 16S rDNA/g of rumen content (Table S1). Archaea were found very soon after birth in almost all lambs, at 5–6 Log_10_ copies of 16S rDNA/g of rumen content. All animals were significantly colonized by Archaea from d21. When lambs got older, the abundance of bacteria stabilized at around 9–10 Log_10_ copies of 16S rDNA/g of rumen content and that of Archaea at around 6–7 Log_10_ copies of 16S rDNA/g.

**Figure 1:**
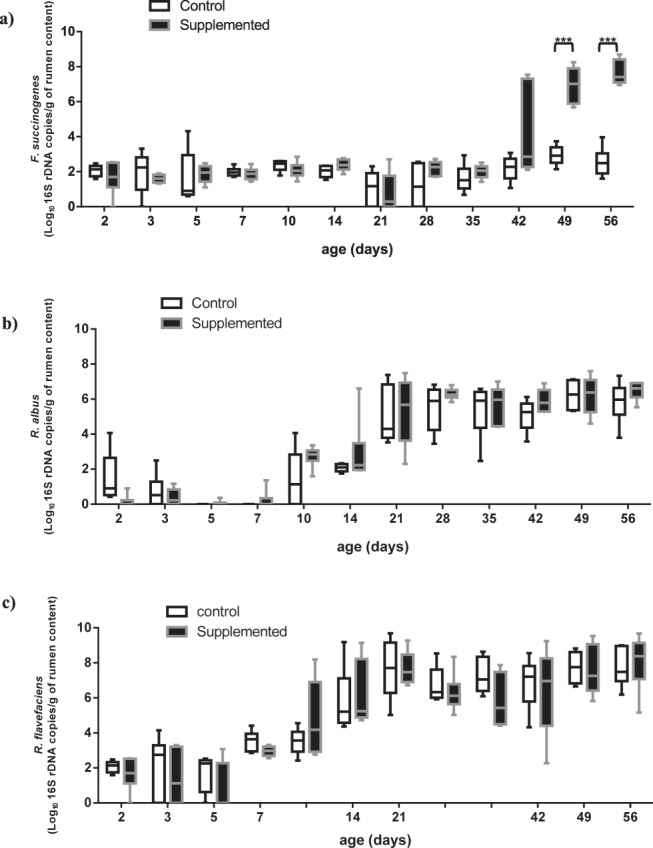
Abundance of *Fibrobacter succinogenes* (**a**), *Ruminococcus albus* (**b**) and *Ruminococcus flavefaciens* (**c**) in the rumen contents (RC) of Control and Supplemented lambs across time measured by qPCR. Effect of supplementation is significant, ***P < 0.0001.

High concentrations of *Prevotella* sp. were found in the lamb rumen very early after birth (Table S1). Individual variability was quite large during the first month, and then much more stable levels were reached in all lambs, on average at 8.7 ± 0.2 Log_10_ 16S rDNA copies/g of rumen content. Kinetics of establishment of *F. succinogenes* and *Ruminococci* in the rumen were different (Fig. 1a–c). While *F. succinogenes* population was quantified at more than 3 Log_10_ copies of 16S rDNA/g of rumen content only after weaning, both species of *Ruminococcus* were found early after birth with population levels greater than 4 Log_10_ copies of 16S rDNA/g of rumen content from the third week of life (Table S1). *R. flavefaciens* was more abundant than *R. albus* at the end of the trial.

In the fecal samples, the total bacterial concentration at d35 and d56 was ~9 Log_10_ 16 S gene copies/g whatever the group. *F. succinogenes* and *R. albus* were under the detection limit of the qPCR (<2 Log_10_ 16S gene copies/g). *R. flavefaciens* was quantified at a higher concentration especially at d56, with quite high variability between animals (5.5 ± 1.5 Log_10_ 16 S gene copies/g). *Prevotella* sp. was quantified at ~8 Log_10_ 16 S gene copies/g and Archaea abundance ranged between 4.5 and 5.2 Log_10_ 16 S gene copies/g whatever the group.

#### Quantification of eukaryotes

**Figure 2:**
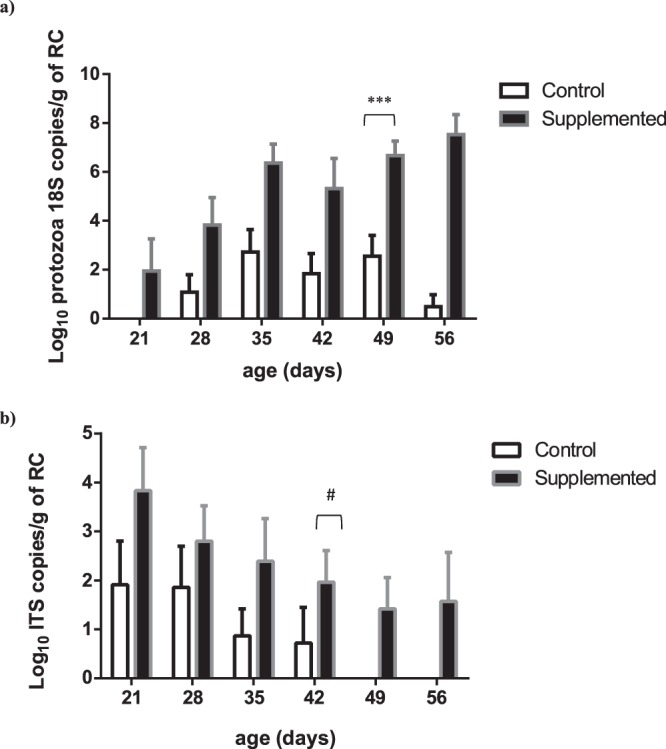
Abundance of protozoa (**a**) and fungi (**b**) in the rumen contents (RC) of Control and Supplemented lambs across time measured by qPCR. Effect of supplementation is significant, ^#^P < 0.10, ***P < 0.0001.

**Figure 3:**
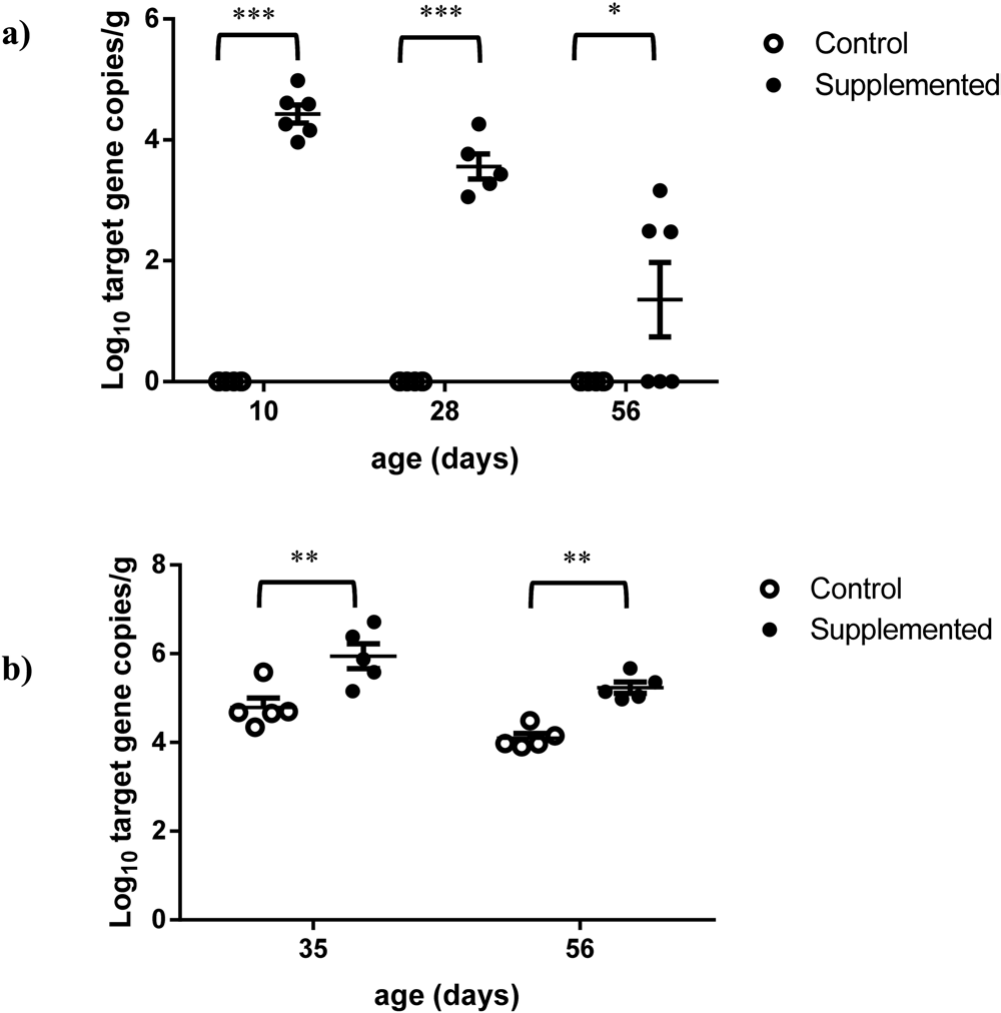
Abundance of *S. cerevisiae* in the rumen contents (**a**) and in the feces (**b**) of Control or Supplemented lambs measured by qPCR. Effect of supplementation is significant, ***P < 0.0001; **P < 0.01; *P < 0.05.

No ciliate cell could be visually identified up to 42 days in any of the rumen samples from Control lambs. In these lambs, protozoa were detected from the first days of age with qPCR (Fig. 2a), but their abundance remained very low and close to the detection threshold (3–4 Log_10_ copies of 18S rDNA/g). Anaerobic fungi were detected only sporadically and at very low concentrations throughout the experiment. Their abundance did not exceed 3–4 Log_10_ copies of ITS/g of rumen content (Fig. 2b). *S. cerevisiae* yeasts were not detected in the rumen of Control lambs throughout the experiment (Fig. 3a) but were quantified in the feces at 4–5 Log_10_ copies of target gene/g (Fig. 3b).

#### Microbiota diversity and taxonomic composition

Using MiSeq, the average of high quality reads per sample was 34,374 ± 17,962, 2,136 ± 3,038, 2,048 ± 3,737 and 2,785 ± 2,824 for Bacteria, Archaea, Eukaryota and Fungi, respectively, among rumen and fecal samples, at different animal ages and in the two studied groups.

**Figure 4:**
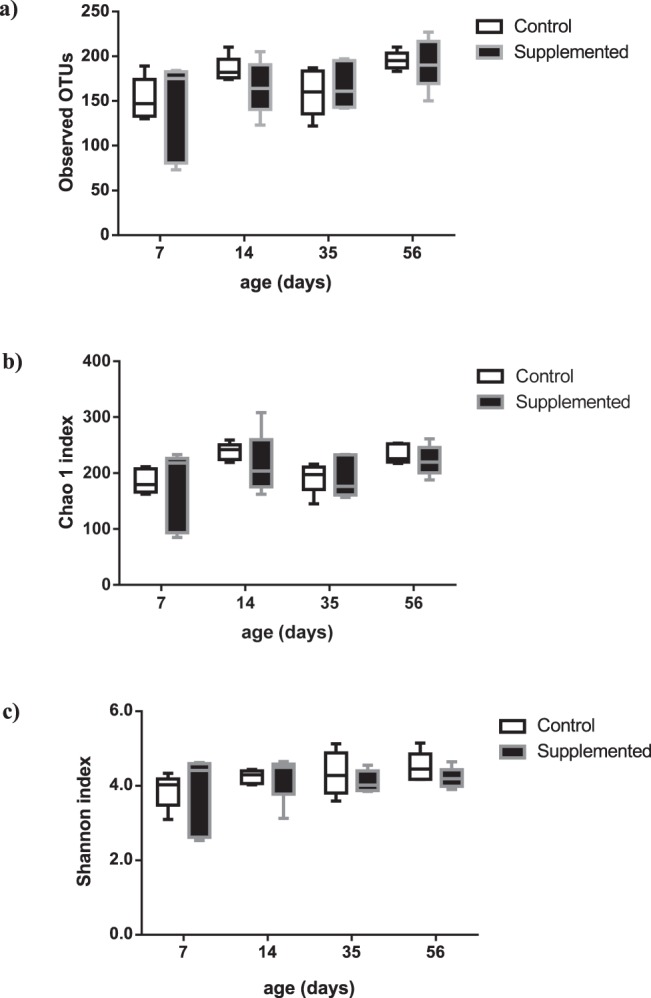
Diversity of Bacteria in the rumen of lambs. Comparison of Observed OTU (**a**), Chao 1 index (**b**) and Shannon index (**c**) of bacterial communities within different ages in Control and Supplemented groups (n = 5 individuals per group).

Numbers of observed OTUs, Chao 1, and Shannon indexes for rumen are shown in Figs. 4 and S2, and for feces on Fig. S3. For Bacteria, number of OTUs and Chao 1 ruminal index significantly increased as the animals matured (Fig. 4, Table S2). For total Eukaryota and Fungi, on the opposite, ruminal diversity measures decreased with time (Fig. S2a,b). There was more Eukaryota richness as measured by OTU number and Chao1 index in the fecal samples than in the rumen samples at the same time points (Fig. S3b). All bacterial diversity indexes were significantly different between feces and rumen (P < 0.05).

**Figure 5:**
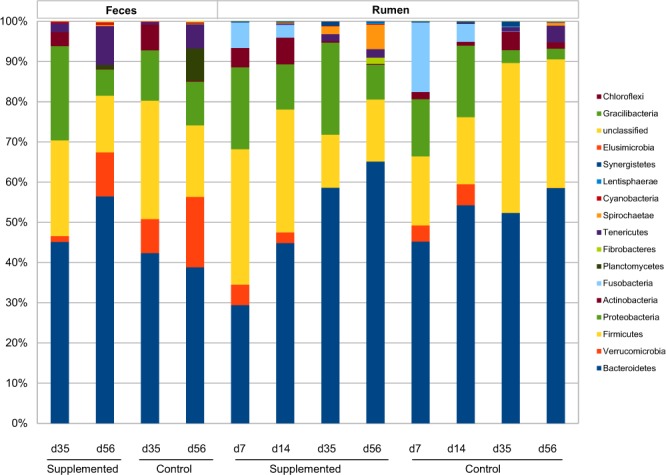
Bar plot representation of mean bacterial composition in the rumen and in the feces at the phylum level for Control and Supplemented lambs.

The bacterial community found in the rumen during the first two weeks of life was different from that found after two weeks. Overall, 16 phyla were identified and the proportion of unclassified sequences was very low (Fig. 5). Bacteroidetes and Firmicutes were the two dominant phyla, Bacteroidetes representing 30–60% of bacterial sequences, and Firmicutes representing 12–30%. The Verrucomicrobia phylum was found in the rumen only during the first two weeks of life, with almost all of the sequences affiliated to the genus *Akkermansia*. The Proteobacteria phylum was the third most abundant phylum; it was particularly abundant before two weeks of life in the rumen. Sequences affiliated to Fusobacteria and Actinobacteria phyla were retrieved in the rumen of lambs only during the first two weeks of life. Within the Bacteroidetes phylum, *Bacteroidaceae* were abundant during the first 2 weeks of life, but from d35, *Prevotellaceae* represented the most abundant family with 30–50% of the bacterial sequences (Fig. S4). Members of *Lachnospiraceae* family, which *Butyrivibrio fibrisolvens* belongs to, were found from d7 and established progressively in the rumen with increasing proportions as the lambs aged. *Veillonellacaeae*, comprising the lactate utilizers *Megasphaera* and *Selenomonas*, represented ~8–10% of total sequences before two weeks of life and decreased afterwards. Sequences affiliated to *Ruminococcaceae* were found in the rumen from d7 at less than 2–3%, but afterwards represented up to 10% of the total bacterial sequences.

Overall, families linked to aerobic or facultative anaerobic metabolism (i.e. *Enterobacteriaceae, Streptococcaceae, Fusobacteriaceae, Pasteurellaceae, Neisseriaceae*) were more abundant before d14 and strict anaerobes developed later on.

Bacteroidetes and Firmicutes were also the two main phyla in the feces, but the Verrucomicrobia and the Proteobacteria phyla represented up to 15–25% of the fecal sequences (Fig. 5). Within the Bacteroidetes phylum, *Bacteroidaceae* was the most dominant family at d35 and d56 (Fig. S4). Sequences belonging to *Ruminococcaceae* were found in the feces in greater proportions than in the rumen. Members of *Desulfovibrionaceae* family were retrieved in all fecal samples, as well as *Campylobacteraceae* and *Enterobacteriaceae*.

**Figure 6:**
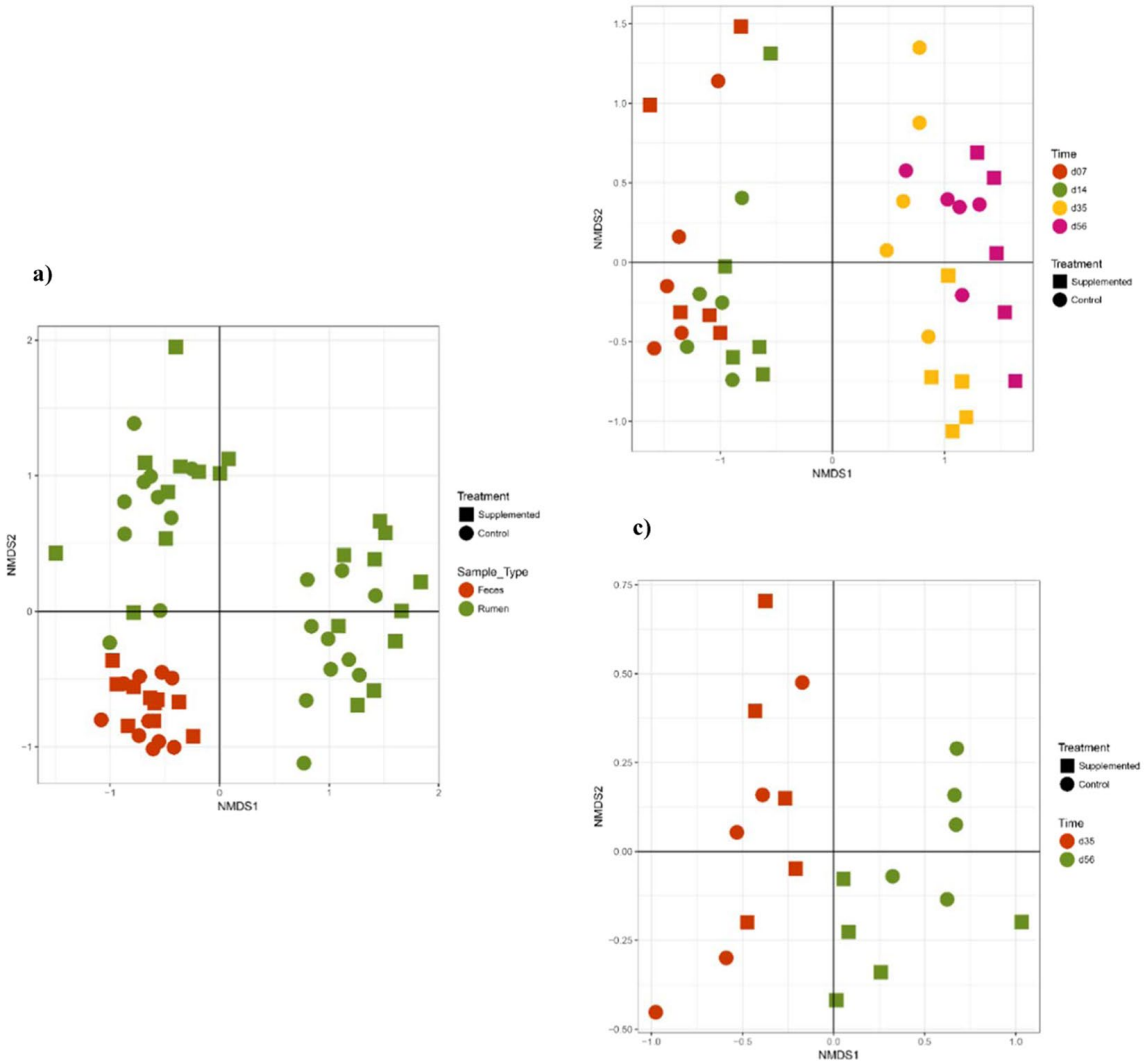
nMDS representations of microbiota according to digestive compartment (**a**), or to age of lambs in the ruminal (**b**) or fecal samples (**c**).

Bacterial community OTU comparisons by non-metric multi-dimensional scaling (nMDS) of each group using the Bray-Curtis similarity metric revealed that the samples clustered together according to the origin of sample (rumen *vs* feces) (Fig. 6a), and, within the same kind of sample, to a particular age, microbiota from d7 and d14 being clearly separated from those from d35 and d56 (Fig. 6b,c). This suggests that there is a time-dependent variation in the bacterial composition in the rumen.

Regarding eukaryotes, a much smaller number of reads was obtained in rumen samples collected before weaning (not shown). This was not surprising as rumen eukaryotes, represented by fungi and ciliate protozoa, are much less abundant than bacteria. In the rumen of Control lambs, a small proportion of sequences were affiliated to Zygomycota (1 OTU) and Basidiomycota (1 OTU) at d7. Neocallimastigomycota represented ~8% of the total sequences at d14 but were not detected at d35 and very weakly at d56 (Fig. S5a). All the sequences in the Ciliophora phylum were assigned to order Litostomatea, family *Trichostomatia*, and genus *Entodinium* (Fig. S5a).

In the feces, the composition of the eukaryotic community was almost exclusively represented by Ascomycota phylum, except at d56 where Ciliophora represented 50% of the sequences (Fig. S5a). At the class level (not shown), four classes of Ascomycota were retrieved, among which *Saccharomycetes* were the most abundant both in fecal and ruminal samples, in almost all samples across time except in fecal samples of the Control group at d56.

Internal Transcribed Spacer (ITS) sequencing was performed in order to better differentiate the fungal communities. The number of sequences was low, which led to a small number of identified OTUs, and in some samples no sequence was retrieved. This was expected as the fungal communities within the rumen or the feces are known to be very under-dominant among the microbiota. At the phylum level (Fig. S5b), rumen samples harbored sequences affiliated to the Ascomycota, which were largely dominant in all samples whatever time and group. Basidiomycota related reads were also found especially after d35, which were very probably linked to contaminations from the hay and the straw distributed to the lambs. After removing these sequences, the fungal community was almost exclusively composed by Ascomycota, at d7 and d14. In the fecal samples (not shown), Ascomycota were also largely dominant.

Regarding Archaeal sequences in the rumen samples, *Methanobrevibacter* appeared as the most dominant genus, with 60–100% of affiliated sequences whatever the age of lambs (data not shown). Ten to twenty percent of the sequences were related to *Methanomassilicoccaceae* at d35 and d56 whatever the group, with different OTUs found. In the feces, the genus *Methanobrevibacter* appeared also as the most dominant genus in the samples (at d35 and d56) and a small proportion of Methanomassilicoccales (less than 5%) was retrieved.

#### Fibrolytic potential of the rumen microbiota

**Figure 7:**
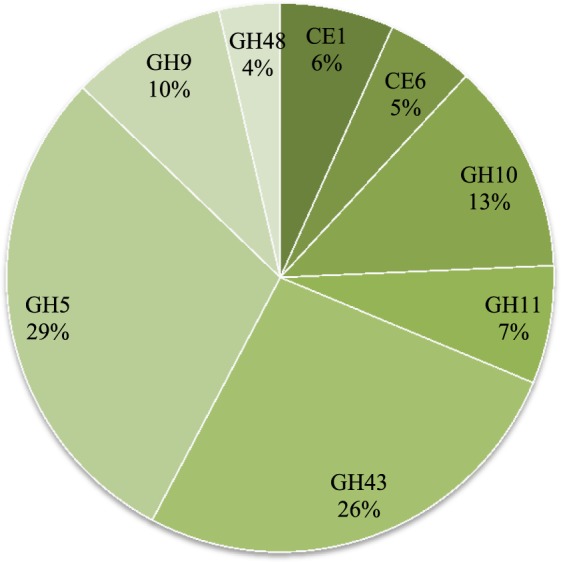
Proportions of GH/CE genes detected in the rumen contents of the lambs (both groups together) at d56 using the FibroChip.

The rumen samples of three animals from each group at d56 were analysed with the FibroChip. Proportions of detected genes according to their carbohydrate active enzyme (CAZyme) family were determined for the 6 lambs (Fig. 7). All the targeted families were detected in the rumen of these young lambs. GH5 and 43 families were largely predominant. We also classified the CAZyme genes according to the genus they belong to (Fig. S6). As expected, genes belonging to *Fibrobacter* and *Ruminococcus* which are two major fibrolytic genera in the rumen, accounted for a large portion of the total CAZyme gene pool. *Bifidobacterium* CAZyme genes were very abundant, mostly for GH5 and GH43 genes. Other bacterial genera such as *Bacteroides*, *Cellulomonas* or *Cellulosilyticum* represented important members of the fibrolytic community (11, 12 and 5% of the total CAZyme gene pool, respectively). Five percent of the total CAZyme gene pool belong to *Prevotella* genus (GH5-10 and 43), and ~ 6% belong to rumen fungi and protozoa.

### Impact of live yeast supplementation on microbial abundance, diversity, taxonomic composition and fibrolytic potential

#### Abundance of prokaryotes by qPCR

There was overall no difference in total bacteria and Archaea ruminal and fecal concentrations between groups. Lambs supplemented with the probiotic combination were colonized by higher numbers of *F. succinogenes* from d35 (Fig. 1a, Table S1). The difference between Control and Supplemented group was overall highly significant (effect of supplement, P < 0.0001), with a significant interaction with time (P < 0.0001) as the effects were mostly measured from d42 and even stronger at d49 and d56 (mean concentration were for Control and Supplemented group respectively, of 2.9 ± 0.2, *vs* 7.0 ± 0.4 Log_10_ copies of 16S rDNA/g of rumen content at d49 and of 2.6 ± 0.3 vs 7.7 ± 0.3 Log_10_ copies of 16S rDNA/g of rumen content at d56).

No significant change in *Ruminococcus albus* or *R. flavefaciens* abundances was observed (Fig. 1b,c), although a numerical increase in rumen colonization by *R. albus* up to weaning (Table S1), and a numerically greater fecal concentration of *R. flavefaciens* were noticed with the supplementation (5.0 ± 1.5 *vs* 6.1 ± 1.4 Log_10_ copies of 16S rDNA/g of rumen content for Control *vs* Supplemented groups, respectively).

#### Abundance of eukaryotes

Microscopic enumeration of protozoa was successful only in the Supplemented group, all lambs of this group harboring ciliates at days 49 and 56 with concentrations ranging between 5 and 6 × 10^5^ cells/mL. The massively dominant morphology was that of small ciliate entodiniomorphs, probably *Entodinium sp*. With qPCR, greater protozoa concentrations were found in the Supplemented group compared to Controls (P < 0.0001), with a significant interaction with time (P < 0.0001) as the effect started from d35. At day 56 there was almost a 5 Log_10_ difference between Supplemented and Control groups (Fig. 2a, Table S1). Furthermore, higher fungal concentrations were measured by qPCR in the Supplemented group than in Control (P < 0.01) (Fig. 2b, Table S1).

Contrary to Control lambs, in Supplemented lambs *S. cerevisiae* yeasts were detected and quantified at ~4 Log_10_ target gene copies/g of rumen content (effect of yeast Supplement was significant, P < 0.0001) with a decrease in concentration across time (P < 0.0001) (Fig. 3a). Their fecal concentrations were significantly higher (P < 0.01 at d35 and P < 0.0001 at d56) in the Supplemented group compared to Control (Fig. 3b).

#### Microbial diversity and taxonomic composition

In the rumen, the bacterial diversity indexes were not different between groups (Fig. 4). However, observed Eukaryota OTU number and Chao1 index as well as Fungi diversity indexes were increased in the rumen of the Supplemented lambs (Fig. S2a,b and Table S2). In the fecal samples, greater diversity measures were also observed for Eukaryota with microbial supplementation (Fig. S3b and Table S3). Beta diversity analysis using Bray Curtis similarity metric did not show any different clustering between microbiota from Supplemented and Control groups (Fig. 6).

Among the phylum Actinobacteria, *Bifidobacteriaceae* related sequences were exclusively recovered in the Supplemented group in early life (Fig. S4). *Lactobacillaceae* were particularly abundant in the rumen at d7 in the Supplemented group compared to Control. Sequences affiliated to *Fibrobacteraceae* were found only in the rumen of Supplemented lambs at d56 (Fig. S4), in rather low proportions (less than 5% of relative abundance).

Between 8 and 23 bacterial OTUs were differentially abundant in the rumen (P < 0.05) according to the group (Table S4). In the d7 samples, the most important difference in abundance was observed for the only OTU affiliated to the genus *Snodgrassella*. With the live yeast based supplement, there was 10 Log_2_-fold more sequences related to this genus than without. OTUs corresponding to *Megasphaera* (OTU000007), *Bifidobacterium* (OTU000015), and *Butyricimonas* (OTU000027) were also significantly more abundant in Supplemented lambs. OTU 000158 related to *Desulfovibrio* genus and 4 OTUs belonging to *Bacteroides* genus were decreased in Supplemented lambs compared to Controls. In the d14 samples, only 8 OTUs were affected by the treatment; the decreased abundance of *Desulfovibrio* was still observed but not on the same OTU. In the d35 samples, the most relevant observation was that *Succinivibrio*/*Succinivibrinoaceae* UGC002 OTUs abundances were strongly increased with the live yeast supplement and that *Snodgrassella* OTU abundance was still increased. In the d56 samples, one OTU affiliated to the genus *Fibrobacter* was much more abundant (~10 Log_2_ fold more reads) in the presence of the live yeast combined with yeast metabolites. In the feces, no differentially abundant OTUs were found in Supplemented lambs compared to Controls (Table S4).

Regarding Archaea taxonomic composition, we noticed few changes according to supplementation. A small percentage of sequences was affiliated to *Methanosphaera* at d35 and d56 only in the Supplemented group.

In the Supplemented lambs, we observed changes in the eukaryotic community composition at the phylum level (Fig. S5a), as from d14, sequences affiliated to Ciliophora were retrieved, their proportion increasing afterwards to represent more than 90% of the sequences at d56. Also, Neocallimastigomycota were found at d14 (~12% of the sequences), at d35 (25% of the sequences), and at d56 (3–5%).

Only a very few eukaryotic OTUs were differentially abundant between groups (Table S5). However, there was a significantly higher abundance (P < 0.05) of two OTUs (cluster 1 and cluster 28) affiliated to Saccharomycetales in rumen samples of Supplemented animals compared to Controls, at d7 and d14, and of one OTU (cluster 1) in fecal samples at d56. In case of Supplemented *vs* Control lambs, the fold change for cluster 1 was clearly greater than for cluster 28. No significant difference between groups could be found for the *Entodinium* related sequences, but we observed 6.6 to 6.9 Log_2_ fold more reads at d35 and d56 respectively in the rumen of Supplemented lambs.

Chytridiomycota, the phylum to which belong rumen anaerobic fungi, accounted for 7–8% and ~38% of the fungal ITS sequences in the Supplemented group at d35 and 56 respectively, whereas this phylum was almost not detected in the Control group (Fig. S5b). All the sequences were affiliated to *Caecomyces* genus.

#### Fibrolytic potential

**Figure 8:**
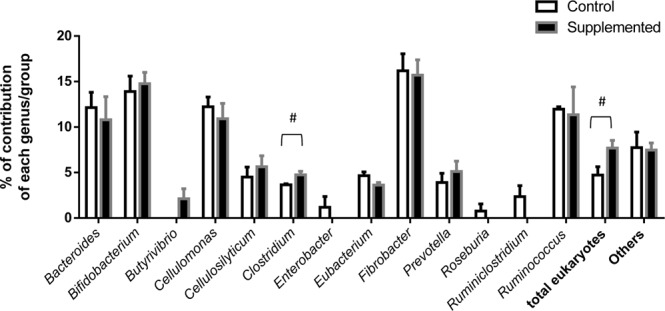
Effect of Supplement on relative contributions of each genus or group to the CAZyme gene pool detected by the FibroChip. Effect is significant, ^#^P < 0.10.

The application of the Fibrochip allowed to show differences in fibrolytic potential according to the yeast supplementation. Indeed, with the microbial supplement a slightly but significantly greater proportion (7.5% *vs* 7.0% in Supplemented *vs* Control, P < 0.05) of the GH11 family was observed and overall, a numerically greater proportion (59.0 *vs* 55.5% in Supplemented *vs* Control) of hemicellulase genes (GH10-GH11-GH43-CE1-CE6) and lower proportion (41.0 *vs* 44.5% in Supplemented *vs* Control) of cellulase genes (GH5-GH9-GH48) were measured. Looking at proportions of the CAZyme genes within each microbial group or genus (Fig. 8), there was a numerically higher proportion of eukaryotic CAZyme genes in the Supplemented group (7.7%) than in the Control group (4.7%, P = 0.07). Only genes from GH5, GH9, GH11 and CE1 were detected as belonging to this Eukaryota microbial group. In the Supplemented group there was also an increase in the global contribution of members of the *Clostridium* genus targeted by our microarray in the pool of CAZyme genes (P = 0.053), a numerical increase in proportion of CAZyme genes belonging to *Butyrivibrio* (P = 0.12) and a numerical decrease in *Ruminiclostridium* representatives (P = 0.12).

Looking into more detail on the detected GH/CE genes that belong to eukaryotes, as said earlier an overall greater contribution of this group with the live yeast and yeast metabolites supplementation was measured (P = 0.07), but no statistical difference was found when considering each family. However, the proportion of GH5, GH9 and CE1 genes increased 2.1 fold, 3.5 fold, and 1.6 fold, respectively, in Supplemented lambs compared to Controls. The increase was the clearest for fungal GH5, GH9 and CE1 and for protozoal GH5.

## Discussion

In our study, lambs were reared in good, well controlled environmental and sanitary conditions, and mortality rate and growth parameters were comparable to what is generally observed in the experimental farm. The variations observed in pH values between individuals might be due to differences in the set-up of fermentative activities during the first month in link with increasing consumption of solid feed, then to maturation of the saliva production system that helped to stabilize rumen environment, and to changes in transit time. Similar pH variations have been reported previously^19^. VFA concentrations measured at early age and the acetate/propionate/butyrate ratios were in agreement with those found in other papers^10^.

Our main objective here was to monitor composition and diversity of the establishing microbiota in the lamb gastro-intestinal tract across time, and to study the impact of a microbial additive distributed from the early age.

### Microbial succession across time

Our data show that the rumen and fecal microbiota composition changed as the animal aged. In the rumen, diversity increased with time, in accordance with previous reports on young ruminants^20^ (lambs, goat kids, calves). *Bacteroidaceae* and *Porphyromonadaceae* were present in the rumen especially before 2 weeks of life, which agrees with data on newborn calves^1,6^. There was a clear change after 2 weeks of age, as strictly anaerobic microbes, representative of the functionally important ruminal communities (*Prevotellaceae*, *Ruminococcaceae*, *Lachnospiraceae*, Clostridiales, *Succinivibrionaceae*) became dominant upon facultative anaerobes (*Enterobacteriaceae, Streptococcaceae, Fusobacteriaceae, Pasteurellaceae, Neisseriaceae*), which is also in accordance with changes in fermentation capacity of the rumen ecosystem. *Snodgrassella* genus has been described previously in the pre-weaning lamb rumen^21^. Representatives of this genus (*S. alvi*) have been isolated from the gut of honey bee, belong to the core microbiome at the ileum level and are thought to provide protection against gut pathogens^22,23^. Our and previous results suggest that *Snodgrassella* may participate to the early shaping of the ecosystem.

In the rumen from d35, *Prevotellaceae* represented the most abundant family with 30–50% of the bacterial sequences. However, qPCR quantification targeting *Prevotella* sp. 16S rRNA gene showed that the abundance of this genus was already high very early after birth (7–9 Log_10_ copies of target gene/g of rumen content). We could suspect that the primers targeting *Prevotella* sp. were not very specific and that members of the *Bacteroidaceae* family could be quantified too with these primers.

In our study, we did not compare lambs kept with their dams and suckling mother’s milk to lambs early separated from their dams and fed MR, but related literature data suggest that the AMS which was applied here had an impact on microbial dynamics in the gastrointestinal tract. Early studies from Fonty *et al*.^11,12,24^ showed that rumen colonization by cellulolytic bacteria and protozoa was impaired when lambs were kept away from their dams right after birth. In goat kids, rumen microbial colonization and rumen development were negatively affected by AMS^10^. In particular, a marked difference in protozoa colonization was observed between goat kids reared with their dams and isolated MR-fed kids. Furthermore, kids isolated and fed MR had a lower rumen weight from 21 days of age and a significant growth impairment. Jami *et al*.^6^ did not detect any *Fibrobacter* related sequences in the rumen of calves before 2 months of age and Yang *et al*.^25^ did not report them either in the rumen of lambs fed MR after 38 days of life. Altogether, these and our data indicate that ciliate protozoa and the cellulolytic bacterial species *Fibrobacter succinogenes* cannot be established properly in the rumen of the lambs under AMS conditions. In our study, we observed also a very sporadic presence of rumen anaerobic fungi which were almost not detected anymore after 42 days of life.

In our lambs, the delay for establishment of those microbial populations was considerable, indicating that these microorganisms probably need specific ecological factors to be able to colonize the rumen, as suggested earlier^12,24^, factors which were not present in the rumen of lambs fed with artificial milk during the first month. *F. succinogenes* has a paramount importance in plant cell wall polysaccharide degradation^26,27^ and rumen protozoa and fungi produce a wide range of fibrolytic enzymes^28–30^; it would thus be possible that this defect impairs fiber digestion efficacy. Compared to *Fibrobacter*, *Ruminococcus* seemed less sensitive to ecological conditions encountered in the rumen of our lambs. *F. succinogenes* could then be proposed as a relevant microbial marker of rumen development. As *F. succinogenes* and *Ruminococci* compete for adhesion to cellulose^31^ as well as for cellulose degradation^32^, the fact that *F. succinogenes* was unable to colonize the rumen early after birth could be favorable to *Ruminococci* establishment. *Ruminococcus flavefaciens* and *R. albus* could be detected early after birth, but at much lower concentrations during the first ten days of life than what was found in young ruminants kept with their mothers^12^. This suggests again that AMS did impair rumen microbial colonization pattern. The proportion of *Ruminococcus* genus increased later in life to reach 1–10% of the total bacteria concentration, probably influenced by solid feed intake. In the feces of our lambs, we were able to find great proportions of sequences (~5–20%) related to *Ruminococcaceae* family and quantify noticeable levels of *R. flavefaciens*, suggesting that this group would play a role in complex carbohydrate digestion in the hindgut. Indeed, together with other bacterial genera, *Ruminococcus* has been identified as an important short chain fatty acid contributor in the hindgut of dairy calves^8^. In the fecal samples, the presence of members of Bacteroidetes, Fimicutes and Proteobacteria as dominant phyla are in agreement with other studies^1,33^ and the absence of Fibrobacteres related sequences was also in accordance with the fact that this phylum would rather be found in the foregut^34^.

Our work provided also an insight in diversity of Archaea in the newborn lamb. At the genus level, *Methanobrevibacter* appeared the most dominant genus in rumen samples at early ages, which was also found in previous studies^4,20,35^. Methanomassilicoccales accounted for more than 10% of the total reads at d35 and 56 in both groups of animals but at d7 in Control group, 40% of sequences were actually affiliated to this order. Given the capacity of Methanomassilicoccales to use a wider range of electron donors than hydrogen, such as methylated compounds (methanol, methylamines)^36^, we could hypothesize that hydrogen availability would be reduced in these lambs which may promote development of this Archean group. However, more research is needed to confirm this hypothesis.

### Effect of the combination of live yeast and yeast metabolites

With a small number of animals, a short experimental period, and given the inter-individual variability, no particular growth benefit was observed upon the distribution of the combined yeast product. Very good farm cleanliness and staff dedicated to animal care might explain why no benefit of the yeast additive was observed in limited stress conditions and in the absence of any disease, as also highlighted by Alugongo *et al*.^37^.

In this study, our main objective was to promote earlier establishment of key functional microbial communities in the rumen and the lower gut of newborn ruminants isolated from their dams early from birth and reared in AMS. To achieve this goal, we chose to combine a live yeast strain, which has been already effective in enhancing fiber degradation and stabilizing rumen pH in different ruminant species^38,39^ including young lambs^16,17^, to selected yeast metabolites that would supply nutrients, vitamins or growth factors for the first steps of microbial establishment. Yeast extracts, yeast fermentation metabolites or yeast cultures have been used with the potential to promote rumen digestion and fermentation^40^. Overall, the combination product used in this study induced changes in microbiota diversity and composition. In particular, the yeast additive allowed ensuring a better establishment of ciliate protozoa, and of *F. succinogenes* in the rumen. Furthermore, an enrichment of sequences affiliated to beneficial genera, i.e. the lactate utilizer *Megasphaera*, butyrate producer *Butyricicoccus*, or health related genus *Bifidobacterium*, was observed. Our results suggest a more functional rumen with key microbial species established earlier in life, which would be of importance during the first weeks of age up to weaning, favoring microbial resilience in case of stress, or increasing ecosystem resistance to pathogen colonization. The increased eukaryotic and fungal alpha diversity in the presence of the yeast supplement could be, at least in part, due to the activity of the *S. cerevisiae* strain in the animal GIT. Like other probiotics, *Saccharomyces cerevisiae* is generally not considered as an autochthonous species in ruminant gastro-intestinal tract, perhaps because of unfavorable physico-chemical conditions and a competitive environment with microbial indigenous communities that are well-adapted to the historical conditions within each rumen, which would prevent consistent niche colonization^41^. However, this species could be seen as a nomadic yeast able to survive in various environments^42^. It is likely that live yeasts which enter the rumen would respond to environmental stressors, e.g. anaerobic conditions, strong competition for nutrients, high concentrations of short chain fatty acids, and this adaptive response to stress could participate to the observed benefits. The oxygen-scavenging capacities described for this strain^43^ could even be considered as promoting unoccupied niche filling as the massive majority of rumen microbial populations are strict anaerobes. It should deserve more research to better understand how the live yeast react to rumen environment, and if metabolic particularities can explain the observed benefits.

Our data are in accordance with previous work which showed that distribution of the same strain of *Saccharomyces cerevisiae* to young lambs kept with their dams, accelerated ciliate colonization kinetics in the rumen and led to higher cellulolytic bacteria concentration^16^.

Beneficial effects of yeasts on ciliate protozoa establishment might be linked to early interactions between rumen microbiota and yeasts but a direct effect is not excluded at this stage, through live yeast metabolic activity or through the supply of important nutrients provided by the combination with yeast metabolites. The yeast based supplement showed some effect on rumen fungi, as shown by qPCR results as well as higher diversity and numerically greater proportions of Neocallimastigomycetes. The yeast strain used in this study has already shown promoting effects on rumen fungi, increasing fiber colonization *in vivo*^38^. The modes of action are not clear yet but we could suspect some effect on the rumen environment, such as reinforcement of strict anaerobic conditions, or provision of specific nutrients such as B vitamins which are known to be essential for zoospore germination^38,43^.

The most marked differences between Control and Supplemented group were observed after 2 weeks-one month of age. Thus, it is probable that the positive effects had occurred through the supplemented pellets which are digested into the rumen, rather than through the supplemented MR, which is directed into the abomasum without entering the rumen. However, the concentration of *S. cerevisiae* measured by qPCR in the rumen of Supplemented lambs was the highest at d10, where milk still represented the major feed, as concentrate was only offered by d8. Amplicon sequencing data were in line with this result as *Saccharomyces* related OTUs were recovered in the rumen at d7 and d14. In our study, lambs had also access to live yeast through MR, and although milk does not generally enter the rumen, it is possible that rumen inoculation had occurred through little milk passage because of immaturity of esophageal groove closure mechanism. Passage of milk into the rumen has been commonly reported in very young animals and would promote lactic acid bacteria development, which could be consistent with the higher proportions of *Lactobacillaceae* and *Bifidobacteriaceae* related sequences measured. Furthermore, it has been shown that live yeast can survive to gastro-intestinal conditions^17^, and is recovered in the feces, which was actually the case here. As lambs are in contact with fecal material that accumulates in the straw bedding, it cannot be excluded that early rumen inoculation by consumption of fecal material might occur also through this route, and even low concentrations would induce substantial effect within the still immature rumen microbial community. Indeed, there is growing evidence that rare microbes may be neglected key species regulating the functioning of different microbial environments^44^. In the rumen, it is noteworthy that anaerobic fungi are present at low concentrations compared to bacteria (10^4^–10^5^ fungal zoospores/mL of rumen fluid) however they play a primary role in plant cell wall degradation as Comtet-Marre *et al*.^45^ showed, using a metatranscriptomic approach, that fungal sequences would contribute for up to 10% to rumen cellulase (GH9 and GH48) and hemicellulase (GH11) transcripts.

In the fecal samples, both amplicon sequencing data and qPCR results did not show significant changes in microbiota composition or abundance with the live yeast supplement. However, substantial amounts of fecal samples could only been obtained after 35 days of age so we might have missed any effect at earlier age.

### Insight into fibrolytic potential of the rumen microbiota, and impact of the yeast supplement

We applied for the first time a custom-made DNA microarray targeting genes involved in fiber degradation in the rumen in order to evaluate the fibrolytic potential of rumen microbiota in young lambs and investigate the impact of the combination of live yeast probiotics and yeast metabolites. DNA microarrays represent suitable tools for studying complex microbial ecosystems as they allow to analyze a large number of genes and samples at the same time, and they are generally more sensitive than sequencing approaches, as they can detect genes belonging to under-dominant members of the microbiota^46,47^.

We observed that GH5 and GH43 were the most represented CAZyme genes in the rumen of our lambs. This result is in accordance with a gene expression study carried out in adult cows using the same microarray^47^, and these two families were also among the most represented gene families retrieved in recent metagenomic or metatranscriptomic studies in cows^45,48–50^. Important GH43 gene numbers have also been found in the genome of fibrolytic bacteria^51,52^. This reinforces the potential key role of GH5 and GH43 CAZymes in cellulose and hemicellulose degradation.

Our microarray results suggest that *Bifidobacterium* genus could play an important role in fiber digestion at early age, as 14% of the genes of the 8 targeted CAZyme families (mostly of GH5 and GH43) actually belong to this genus. *Bifidobacterium* could be important in the degradation of short dietary polysaccharides but also of mucopolysaccharides that are released from the maturing rumen epithelium, as these bacteria are known to possess numerous activities involved in mucin degradation^53^.

*Bacteroides*, *Cellulomonas* or *Cellulosilyticum* appeared to represent important members of the fibrolytic community; these genera are still very poorly characterized in the ruminant animal but recent metatranscriptomic data suggest they are involved in plant cell wall degradation^45^. *Prevotella* sequences represented almost 50% of 16S sequences in the lambs while the FibroChip detected only 5% of the targeted CAZyme gene pool affiliated to this genus (GH5, 10 and 43). This suggests that *Prevotella* detected in the young animals would probably not be greatly involved in cellulose or hemicellulose degradation but more on pectin, starch and protein metabolism. No difference in *Fibrobacter* contributions to CAZyme gene pool was measured between groups, which was a bit surprising when compared to our other data. Indeed, with the qPCR we measured that the abundance of *Fibrobacter succinogenes* was significantly higher with the probiotic treatment. However, the *Fibrobacter succinogenes* species is taxonomically divided into different subgroups^54^ and the FibroChip does not target the CAZyme genes from all these subgroups, which may have been actually quantified with qPCR as we targeted the 16S rRNA gene.

Data on the effect of microbial supplementation on the proportion of GH11 genes and hemicellulase genes (i.e. CE1, CE6, GH10, GH11, and GH43) would suggest a greater hemicellulolytic potential. Hemicelluloses represent a broad family of plant cell wall polysaccharides, therefore a greater hydrolytic potential would be beneficial for adaptation of the microbial ecosystem to dietary diversification at weaning. This hypothesis has to be further investigated.

Only genes from GH5, GH9, GH11 and CE1 were detected as belonging to eukaryotes. There was a trend for a higher proportion of eukaryotic CAZyme genes in the Supplemented group. Although these differences were not statistically significant, qPCR and amplicon sequencing data on the same samples gave corroborating results. Overall, we suggest that the live yeast based supplement favors the establishment of fibrolytic protozoa and fungi. This is of particular importance given the recent data highlighting the underestimated role of these populations on fiber degradation^45,47^.

In conclusion, our data suggest that the use of live yeast based feed additive improves microbial colonization in the maturing rumen, with a potentially more specialized ecosystem towards efficient fiber degradation, which suggests in turn a possible positive impact on digestive efficiency. However, more research is still needed to help to decipher the microbial dynamics in the young ruminant gastro-intestinal tract and to precise whether the benefits that have been observed with the probiotic supplementation in young animals can be translated into improved digestive function and health later in life.

## Material and Methods

### Animals and diets

Sixteen lambs (*Ovis aries*, Romane breed) were used for this study. They were selected at birth and assigned to two groups (Control, Supplemented) which were balanced homogeneously regarding sex and birth weight (minimal birth weight was fixed to 2.5 kg). Twins were systematically separated in different groups. Lambs were kept with their dams at least 12 hours in order to ensure colostrum uptake.

The two pens (Control and Supplemented) were physically separated to ensure that no contact occurred between the groups. Bedding was made of straw. Lambs were bottle fed during the first day, then they were trained to suckle teats (8 teats were proposed per group) until they become enough autonomous. Milk replacer was prepared twice a day by mixing milk powder (Bonilait protéines, Chasseneuil du Poitou, France) with hot water (40 °C maximum). Feed additives were mixed in a minimal quantity of milk until complete dissolution, and then the final quantity of milk was prepared. The Supplement was offered with the morning milk meal. Individual doses of feed additives were prepared in advance. For the Supplemented group, 3 × 10^9^ CFU of *Saccharomyces cerevisiae* CNCM I-1077 (Levucell SC, Lallemand SAS, Blagnac, France) mixed with 0.45 g of a specific combination of yeast metabolites (Lallemand SAS, Blagnac, France) were prepared for one daily individual dose.

When lambs were 8 days old, they received a pelleted concentrate (Table S6) until the end of the experiment (60 days of age), good quality meadow hay and good quality water. Feed additives had been incorporated during the manufacturing process (Moulin de Massagettes, Massagettes, France). For this purpose, we used the microencapsulated form of Levucell SC, i.e. Levucell SC Titan, at a rate of ~6 × 10^6^ CFU/g of feed mixed with the specific preparation of yeast metabolites added at a rate of 1.5 kg/ton of feed.

Total yeast enumeration was performed in experimental concentrates throughout the study (the day of concentrate delivery, and 15d, 30d and 60 days after). Briefly, 30 g of pellet was ground 30 sec in a Waring blender, then suspended in peptone water, ground again for 1 min in the Waring blender, transferred in a stomacher, and homogenized for 1 min in a filter bag. One mL was then collected from the bag and diluted in 9 mL of sterile peptone water. Serial dilutions were performed and plated onto Sabouraud + Chloramphenicol agar Petri dishes (AES Chemunex/BioMérieux, Combourg, France). Plates were incubated during 48 h at 30 °C before colonies could be counted.

The animal trial was conducted at the animal facilities of INRA Herbipôle Experimental Unit UE1414 (Saint-Genès Champanelle, France). Procedures on animals were carried out in accordance with the guidelines for animal research of the French Ministry of Agriculture and all other applicable national and European guidelines and regulations for experimentation with animals (see http://www2.vet-lyon.fr/ens/expa/acc_regl.html for details). The protocol was accepted by the Regional Ethics Committee on Animal Experimentation C2EA-02 with reference number 6895-2016091913586944V3.

### Samples collection

The days of sampling are shown on Fig. S7.

Rumen contents were collected via a stomach PVC tube whose diameter was adapted to the lamb age and morphology. As the first sample was collected at d2, it was not possible to adapt the animals in advance to the stomach tubing procedure. Therefore, contention of lambs was really smooth and the tube was carefully introduced in the mouth of the lamb and pushed gently inside the rumen. The regurgitated digestive contents were retrieved in a sterile tube. The quality of the sample was visually checked (absence of milk, absence of saliva, no trace of blood). During the milk feeding period, lambs were sampled in the morning, after the milk meal, and when lambs consumed solid feed, animals were sampled before morning feeding. Immediately after sampling, pH was recorded and samples were brought back to the laboratory where they were processed. One portion was treated for analysis of volatile fatty acids (VFA) and protozoa enumeration, the other was frozen at −20 °C for microbial analysis with molecular methods. Fecal contents were collected manually in the rectum of lambs and frozen at −20 °C for further analysis.

### Parameters measured

Growth was monitored by weighing lambs at day 0, 10, 20, 42 and 60 of age. Mortality and occurrence of pathologies were recorded. Rumen pH was measured immediately after sampling using a laboratory pH probe. VFA analysis was performed on rumen samples as previously described^55^. Protozoa were enumerated in a Neubauer chamber under a microscope after staining with methyl green solution according to Villot *et al*.^55^.

DNA was extracted from 2 × 250 mg of rumen or fecal contents following Yu and Morrison procedure^56^. DNA yield and quality were determined after Nanodrop 1000 spectrophotometric quantification. DNA extracts were stored at −20 °C until analysis.

Microbial populations were quantified using qPCR method, with specific primer sets and PCR conditions targeting ribosomal RNA genes of total bacteria, protozoa, archaea, the cellulolytic bacteria *Prevotella sp., Fibrobacter succinogenes, Ruminococcus albus, R. flavefaciens*, and the yeast *S. cerevisiae*, and the Internal transcribed spacer 1 (ITS1) of rumen fungi. PCR targets and primers are summarized in Table S7.

Standards were used to determine the absolute abundance of microbial groups, expressed as the Log_10_ number of gene copies per microgram of DNA. For total bacteria, cellulolytic bacteria, and methanogenic archaea, the standard curves were prepared according to Mosoni *et al*.^57^. For protozoa, the standard curve was prepared according to Bayat *et al*.^58^. For each target, a standard curve was prepared from 10^2^ to 10^9^ copies by serial dilution. For *S. cerevisiae* quantification, the standard curve was constructed using DNA obtained from Levucell SC20 commercial product. A PBS-suspension of 10^8^ CFU/g was prepared (total CFU /g in the commercial product was previously checked) and DNA extraction was performed on this suspension. Decimal dilutions of DNA were done to get a range of corresponding cell concentrations of 10^7^ to 10^3^ CFU/g. It was then possible to obtain a standard curve relating yeast cell concentrations and Ct values. *S. cerevisiae* quantification was performed on a limited number of time points (d10, 28 and 56). Efficiency of the qPCR for each target varied between 97 and 102% with a slope from −3.0 to −3.4 and a regression coefficient above 0.95 in accordance with the MIQE guidelines^59^.

For amplicon sequencing, DNA samples were quantified with a Qubit spectrophotometer to adjust concentrations at 20 ng/µL. A volume of 20 µL per sample was sent to the sequencing platform (W.M. Keck Center for Comparative and Functional Genomics at the University of Illinois at Urbana-Champaign, USA). We selected 5 lambs per group for which we had good amounts of DNA for each sampling time considered. The samples analyzed included rumen contents at days 7, 14, 35 and 56 and fecal contents at days 35 and 56. The diversity and composition of rumen/fecal microbiota were studied using the high throughput sequencing Illumina MiSeq (Illumina, San Diego, CA) method (2 × 250 nt paired ends). The primer sets used and rDNA regions targeted are indicated in Table S8. Libraries construction and MiSeq Illumina sequencing were carried out following protocols validated in the W.M. Keck Center for Comparative and Functional Genomics at the University of Illinois at Urbana-Champaign, USA.

Raw reads were first trimmed to eliminate low quality sequences using Trimmomatic v0.32^60^. For the 16S and ITS datasets, the sequences were then clustered into OTUs, cleaned from chimeras and classified using Mothur v1.36.0^61^. For the 18S dataset, sequences were analyzed with the Galaxy supported FROGS pipeline^62^. The databases used for taxonomic affiliation were Silva v123^63^ for Bacteria and Eukaryota, UNITE v99^64^ for ITS and RIM-Db^65^ for Archaea. The OTUs tables were then filtered to eliminate singletons and very low abundant reads: an OTU was retained if present at least 2 times in at least 5 samples. Graphical and statistical analyses were performed on filtered OTUs tables using the R packages ggplot2 and Phyloseq v1.16.2^66^ with the DESeq. 2^67^ method to test for differentially abundant OTUs. P-values were adjusted using the Benjamini–Hochberg procedure^68^. Rarefaction indices and curves were computed using Qiime v1.9.1^69^. To perform all the bioinformatics analyses we used the Genotoul bioinformatics platform Toulouse Occitanie computing and storage resources.

We also used a custom-made DNA microarray, called FibroChip, to analyse the fibrolytic potential of the rumen ecosystem. The tool is described in Comtet-Marre *et al*.^47^. Briefly, the FibroChip targets 392 genes belonging to 8 families of carbohydrate-active enzymes (CAZymes) and present in 41 bacterial, 4 protozoal and 10 fungal species. The targeted genes code for glycoside hydrolase (GH) families 5, 9, 10, 11, 43 and 48 and for carbohydrate-esterase (CE) families 1 and 6. The FibroChip is constituted by 4249 oligonucleotide probes. Here we used the FibroChip to identify the genes that were present in the rumen samples by hybridizing gDNA on the chip. We selected rumen samples from only 3 lambs per group, for which we had both complete qPCR and amplicon sequencing data. The procedures for DNA preparation, labeling (Cy3) and hybridization are described in Comtet-Marre *et al*.^47^. Determination of SNR (signal to noise ratio) for each detected gene was performed according to Comtet-Marre *et al*.^47^. In our experiment, the SNR threshold was set at 2.3.

### Statistical analyses

Graphical representations were performed using GraphPad Prism 6.0 and the effect of supplementation and interaction with time were evaluated using two-way ANOVA and multiple comparisons with Tukey’s adjustment. Statistical significance was determined at a P value < 0.05 and trends discussed when P < 0.10.

### Accession numbers

Sequencing data are available in the BioProject SRA database (https://www.ncbi.nlm.nih.gov/sra/) as PRJNA482962 under the accession number SRP155341. The FibroChip data discussed in this publication have been deposited in NCBI’s Gene Expression Omnibus^70^ and are accessible through GEO Series accession number GSE122256.

## Supplementary information

Supplementary information 

## References

[1] Yeoman, C. J. *et al*. Biogeographical Differences in the Influence of Maternal Microbial Sources on the Early Successional Development of the Bovine Neonatal Gastrointestinal tract. *Sci. Rep*. **8** (2018). 10.1038/s41598-018-21440-8 PMC5816665 29453364

[2] Meale SJ, Chaucheyras-Durand F, Berends H, Guan LL, Steele MA (2017). From pre- to postweaning: Transformation of the young calf’s gastrointestinal tract. J. Dairy Sci..

[3] Guzman, C. E., Bereza-Malcolm, L. T., De Groef, B. & Franks, A. E. Presence of Selected Methanogens, Fibrolytic Bacteria, and Proteobacteria in the Gastrointestinal Tract of Neonatal Dairy Calves from Birth to 72 Hours. *PLoS ONE* **10** (2015). 10.1371/journal.pone.0133048 PMC4505879 26186002

[4] Wang Z (2017). Changes in Metabolically Active Bacterial Community during Rumen Development, and Their Alteration by Rhubarb Root Powder Revealed by 16S rRNA Amplicon Sequencing. Front. Microbiol..

[5] Li RW, Connor EE, Li C, Baldwin Vi RL, Sparks ME (2012). Characterization of the rumen microbiota of pre-ruminant calves using metagenomic tools. Environ. Microbiol..

[6] Jami E, Israel A, Kotser A, Mizrahi I (2013). Exploring the bovine rumen bacterial community from birth to adulthood. ISME J..

[7] Yáñez-Ruiz DR, Abecia L, Newbold CJ (2015). Manipulating rumen microbiome and fermentation through interventions during early life: a review. Front. Microbiol..

[8] Song, Y., Malmuthuge, N., Steele, M. A. & Guan, L. L. Shift of hindgut microbiota and microbial short chain fatty acids profiles in dairy calves from birth to pre-weaning. *FEMS Microbiol. Ecol*. **94** (2018). 10.1093/femsec/fix179 29267960

[9] Malmuthuge N, Griebel PJ, Guan LL (2015). The Gut Microbiome and Its Potential Role in the Development and Function of Newborn Calf Gastrointestinal Tract. Front. Vet. Sci..

[10] Abecia L (2014). Feeding management in early life influences microbial colonisation and fermentation in the rumen of newborn goat kids. Anim. Prod. Sci..

[11] Fonty G, Gouet P, Jouany JP, Senaud J (1983). Ecological factors determining establishment of cellulolytic bacteria and protozoa in the rumens of meroxenic lambs. J. Gen. Microbiol..

[12] Fonty G, Senaud J, Jouany JP, Gouet P (1988). Establishment of ciliate protozoa in the rumen of conventional and conventionalized lambs: influence of diet and management conditions. Can. J. Microbiol..

[13] Mialon, M. M. 3R - Rencontres autour des Recherches sur les Ruminants, http://www.journees3r.fr/spip.php?article4214.

[14] Abecia L, Martín-García AI, Martínez G, Newbold CJ, Yáñez-Ruiz DR (2013). Nutritional intervention in early life to manipulate rumen microbial colonization and methane output by kid goats postweaning. J. Anim. Sci..

[15] Abecia L (2014). An antimethanogenic nutritional intervention in early life of ruminants modifies ruminal colonization by Archaea. Archaea Vanc. BC.

[16] Chaucheyras-Durand F, Fonty G (2002). Influence of a Probiotic Yeast (*Saccharomyces cerevisiae* CNCM I-1077) on Microbial Colonization and Fermentations in the Rumen of Newborn Lambs. Microb. Ecol. Health Dis..

[17] Chaucheyras-Durand F, Fonty G (2001). Establishment of cellulolytic bacteria and development of fermentative activities in the rumen of gnotobiotically-reared lambs receiving the microbial additive *Saccharomyces cerevisiae* CNCM I-1077. Reprod. Nutr. Dev..

[18] Leuschner RGK, Bew J, Bertin G (2003). Validation of an official control method for enumeration of authorised probiotic yeast in animal feed. Syst. Appl. Microbiol..

[19] Rey M, Enjalbert F, Monteils V (2012). Establishment of ruminal enzyme activities and fermentation capacity in dairy calves from birth through weaning. J. Dairy Sci..

[20] Dill-McFarland KA, Breaker JD, Suen G (2017). Microbial succession in the gastrointestinal tract of dairy cows from 2 weeks to first lactation. Sci. Rep..

[21] Liu J, Bian G, Sun D, Zhu W, Mao S (2017). Starter feeding altered ruminal epithelial bacterial communities and some key immune-related genes’ expression before weaning in lambs. J. Anim. Sci..

[22] Kwong WK, Moran NA (2016). Gut microbial communities of social bees. Nat. Rev. Microbiol..

[23] Maes PW, Rodrigues PAP, Oliver R, Mott BM, Anderson KE (2016). Diet-related gut bacterial dysbiosis correlates with impaired development, increased mortality and *Nosema disease* in the honeybee (*Apis mellifera*). Mol. Ecol..

[24] Fonty G, Gouet P, Ratefiarivelo H, Jouany JP (1988). Establishment of *Bacteroides succinogenes* and measurement of the main digestive parameters in the rumen of gnotoxenic lambs. Can. J. Microbiol..

[25] Yang, B. *et al*. Alfalfa Intervention Alters Rumen Microbial Community Development in Hu Lambs During Early Life. *Front. Microbiol*. **9** (2018). 10.3389/fmicb.2018.00574 PMC5881016 29636743

[26] Béra-Maillet C, Ribot Y, Forano E (2004). Fiber-degrading systems of different strains of the genus Fibrobacter. Appl. Environ. Microbiol..

[27] Kobayashi Y, Shinkai T, Koike S (2008). Ecological and physiological characterization shows that Fibrobacter succinogenes is important in rumen fiber digestion - review. Folia Microbiol. (Praha).

[28] Edwards JE (2017). PCR and Omics Based Techniques to Study the Diversity, Ecology and Biology of Anaerobic Fungi: Insights, Challenges and Opportunities. Front. Microbiol..

[29] Béra-Maillet C, Devillard E, Cezette M, Jouany J-P, Forano E (2005). Xylanases and carboxymethylcellulases of the rumen protozoa *Polyplastron multivesiculatum*, *Eudiplodinium maggii* and *Entodinium* sp. FEMS Microbiol. Lett..

[30] Newbold CJ, de la Fuente G, Belanche A, Ramos-Morales E, McEwan NR (2015). The Role of Ciliate Protozoa in the Rumen. Front. Microbiol..

[31] Mosoni P, Fonty G, Gouet P (1997). Competition between ruminal cellulolytic bacteria for adhesion to cellulose. Curr. Microbiol..

[32] Shi Y, Odt CL, Weimer PJ (1997). Competition for cellulose among three predominant ruminal cellulolytic bacteria under substrate-excess and substrate-limited conditions. Appl. Environ. Microbiol..

[33] Huws SA (2018). Addressing Global Ruminant Agricultural Challenges Through Understanding the Rumen Microbiome: Past, Present, and Future. Front. Microbiol..

[34] Zeng Y (2017). Microbial community compositions in the gastrointestinal tract of Chinese Mongolian sheep using Illumina MiSeq sequencing revealed high microbial diversity. AMB Express.

[35] Skillman LC (2004). 16S ribosomal DNA-directed PCR primers for ruminal methanogens and identification of methanogens colonising young lambs. Anaerobe.

[36] Borrel G (2013). Phylogenomic data support a seventh order of Methylotrophic methanogens and provide insights into the evolution of Methanogenesis. Genome Biol. Evol..

[37] Alugongo GM (2017). Review: Utilization of yeast of *Saccharomyces cerevisiae* origin in artificially raised calves. J. Anim. Sci. Biotechnol..

[38] Chaucheyras-Durand F (2016). Live yeasts enhance fibre degradation in the cow rumen through an increase in plant substrate colonization by fibrolytic bacteria and fungi. J. Appl. Microbiol..

[39] Chaucheyras-Durand F, Walker N, Bach A (2008). Effects of active dry yeasts on the rumen microbial ecosystem: Past, present and future. Anim. Feed Sci. Technol..

[40] Poppy GD (2012). A meta-analysis of the effects of feeding yeast culture produced by anaerobic fermentation of Saccharomyces cerevisiae on milk production of lactating dairy cows. J. Dairy Sci..

[41] Weimer PJ (2015). Redundancy, resilience, and host specificity of the ruminal microbiota: implications for engineering improved ruminal fermentations. Front. Microbiol..

[42] Garcia-Mazcorro, J. F. *et al*. Review: Are there indigenous Saccharomyces in the digestive tract of livestock animal species? Implications for health, nutrition and productivity traits. *Animal* 1–9, 10.1017/S1751731119001599 (2019). 10.1017/S1751731119001599 31303186

[43] Fonty G, Chaucheyras-Durand F (2006). Effects and modes of action of live yeasts in the rumen. Biologia (Bratisl.).

[44] Jousset A (2017). Where less may be more: how the rare biosphere pulls ecosystems strings. ISME J..

[45] Comtet-Marre S (2017). Metatranscriptomics Reveals the Active Bacterial and Eukaryotic Fibrolytic Communities in the Rumen of Dairy Cow Fed a Mixed Diet. Front. Microbiol..

[46] Abot A (2016). CAZyChip: dynamic assessment of exploration of glycoside hydrolases in microbial ecosystems. BMC Genomics.

[47] Comtet-Marre, S. *et al*. FibroChip, a Functional DNA Microarray to Monitor Cellulolytic and Hemicellulolytic Activities of Rumen Microbiota. *Front. Microbiol*. **9** (2018). 10.3389/fmicb.2018.00215 PMC5816793 29487591

[48] Hess M (2011). Metagenomic discovery of biomass-degrading genes and genomes from cow rumen. Science.

[49] Dai X (2015). Metatranscriptomic analyses of plant cell wall polysaccharide degradation by microorganisms in the cow rumen. Appl. Environ. Microbiol..

[50] Dai X (2012). Metagenomic insights into the fibrolytic microbiome in yak rumen. PloS One.

[51] Suen G (2011). The complete genome sequence of *Fibrobacter succinogenes* S85 reveals a cellulolytic and metabolic specialist. PloS One.

[52] Suen G (2011). Complete genome of the cellulolytic ruminal bacterium *Ruminococcus albus* 7. J. Bacteriol..

[53] Tailford LE, Crost EH, Kavanaugh D, Juge N (2015). Mucin glycan foraging in the human gut microbiome. Front. Genet..

[54] Neumann AP, Suen G (2018). The Phylogenomic Diversity of Herbivore-Associated *Fibrobacter* spp. Is Correlated to Lignocellulose-Degrading Potential. mSphere.

[55] Villot C, Meunier B, Bodin J, Martin C, Silberberg M (2018). Relative reticulo-rumen pH indicators for subacute ruminal acidosis detection in dairy cows. *Anim. Int*. J. Anim. Biosci..

[56] Yu Z, Morrison M (2004). Improved extraction of PCR-quality community DNA from digesta and fecal samples. BioTechniques.

[57] Mosoni P, Martin C, Forano E, Morgavi DP (2011). Long-term defaunation increases the abundance of cellulolytic ruminococci and methanogens but does not affect the bacterial and methanogen diversity in the rumen of sheep. J. Anim. Sci..

[58] Bayat AR (2015). Effect of camelina oil or live yeasts (*Saccharomyces cerevisiae*) on ruminal methane production, rumen fermentation, and milk fatty acid composition in lactating cows fed grass silage diets. J. Dairy Sci..

[59] Bustin SA (2009). The MIQE guidelines: minimum information for publication of quantitative real-time PCR experiments. Clin. Chem..

[60] Bolger AM, Lohse M, Usadel B (2014). Trimmomatic: a flexible trimmer for Illumina sequence data. Bioinforma. Oxf. Engl..

[61] Schloss PD (2009). Introducing mothur: open-source, platform-independent, community-supported software for describing and comparing microbial communities. Appl. Environ. Microbiol..

[62] Escudié F (2018). FROGS: Find, Rapidly, OTUs with Galaxy Solution. Bioinformatics.

[63] Quast C (2013). The SILVA ribosomal RNA gene database project: improved data processing and web-based tools. Nucleic Acids Res..

[64] Abarenkov K (2010). The UNITE database for molecular identification of fungi–recent updates and future perspectives. New Phytol..

[65] Seedorf H, Kittelmann S, Henderson G, Janssen PH (2014). RIM-DB: a taxonomic framework for community structure analysis of methanogenic archaea from the rumen and other intestinal environments. PeerJ.

[66] McMurdie PJ, Holmes S (2015). Shiny-phyloseq: Web application for interactive microbiome analysis with provenance tracking. Bioinforma. Oxf. Engl..

[67] Love. Moderated estimation of fold change and dispersion for RNA-seq data with DESeq. 2|Genome Biology|Full Text, https://genomebiology.biomedcentral.com/articles/, 10.1186/s13059-014-0550-8. 10.1186/s13059-014-0550-8 PMC4302049 25516281

[68] Benjamini, Y. & Hochberg, Y. Controlling the false discovery rate: a practical and powerful approach to multiple testing. *J R Stat Soc Ser B Methodol* **57** (1995).

[69] Caporaso JG (2010). QIIME allows analysis of high-throughput community sequencing data. Nat. Methods.

[70] Edgar R, Domrachev M, Lash AE (2002). Gene Expression Omnibus: NCBI gene expression and hybridization array data repository. Nucleic Acids Res..

