# Supplementary material for: Supplementation of live yeast based feed additive in early life promotes rumen microbial colonization and fibrolytic potential in lambs
Source: Sci Rep. 2019 Dec 16;9:19216. doi: 10.1038/s41598-019-55825-0 (PMC6914811; doi:10.1038/s41598-019-55825-0)
Supplement: Supplementary file 1 — Supplementary information [file 41598_2019_55825_MOESM1_ESM.docx]

**Supplementary Material**

**Supplementation of live yeast based feed additive in early life promotes rumen microbial colonization and fibrolytic potential in lambs**

Chaucheyras-Durand Fréderique^1,2^, Ameilbonne Aurélie^1,2^, Auffret Pauline^2 #^, Bernard Mickael^3^, Mialon Marie-Madeleine^4^, Dunière Lysiane^1,2^, Forano Evelyne^2^

^1^Lallemand SAS, 31702 Blagnac, France

^2^ Université Clermont Auvergne, INRA, UMR 454 MEDIS, F-63000 Clermont-Ferrand, France

^3^ UE 1414 Herbipôle, INRA Auvergne Rhône Alpes, F-63122 Saint-Genès Champanelle, France

^4^ Université Clermont Auvergne, INRA, VetAgro Sup, UMR 1213 Herbivores, F-63000 Clermont-Ferrand, France

# present address: Ifremer, UMR 241 EIO, Tahiti, French Polynesia

**Figure and Table legends**

Figure S1: Lambs Body weight and rumen pH

Figure S2: Diversity of total Eukaryota (a), Fungi (b) and Archaea (c) in the rumen of lambs.

Figure S3: Diversity of Bacteria (a), Eukaryota (b), Fungi (c) and Archaea (d) in the feces of lambs.

Figure S4: Bar plot representation of mean bacterial composition in the rumen and in the feces at the family level for Control and Supplemented lambs.

Figure S5: Bar plot representation of mean Eukaryota (a) and Fungi (b) composition in the rumen a at the phylum level for Control and Supplemented lambs.

Figure S6: Contribution of the different rumen microbial genera or groups to the CAZyme gene pool detected by the FibroChip (both groups combined).

Figure S7: Experimental scheme and sampling times.

Table S1: Concentrations of the different microbial groups targeted by qPCR (copies of target gene/g of rumen content or fecal content). nd = not detectable (under the qPCR sensitivity threshold); na = not analyzed.

Table S2: Alpha diversity measures in the rumen of the two lamb groups across time (averages of 5 individuals for each group). ns= not significant (P>0.05).

Table S3: Alpha diversity measures in the feces of the two lamb groups across time (averages of 5 individuals for each group). ns= not significant (P>0.05).

Table S4: Differential OTU analysis for Bacteria in the rumen and the feces of Control and Supplemented lambs. (Excel file)

Table S5: Differential OTU analysis for Eukaryota in the rumen and the feces of Control and Supplemented lambs. (Excel file)

Table S6: Composition of the concentrate fed to lambs.

Table S7: qPCR targets and primers.

Table S8: Primers used for Illumina MiSeq.

**a)**

**b)**

Figure S1

**a)**

**b)**

**c)**

Figure S2

**d)**

**c)**

**b)**

**a)**

Figure S3


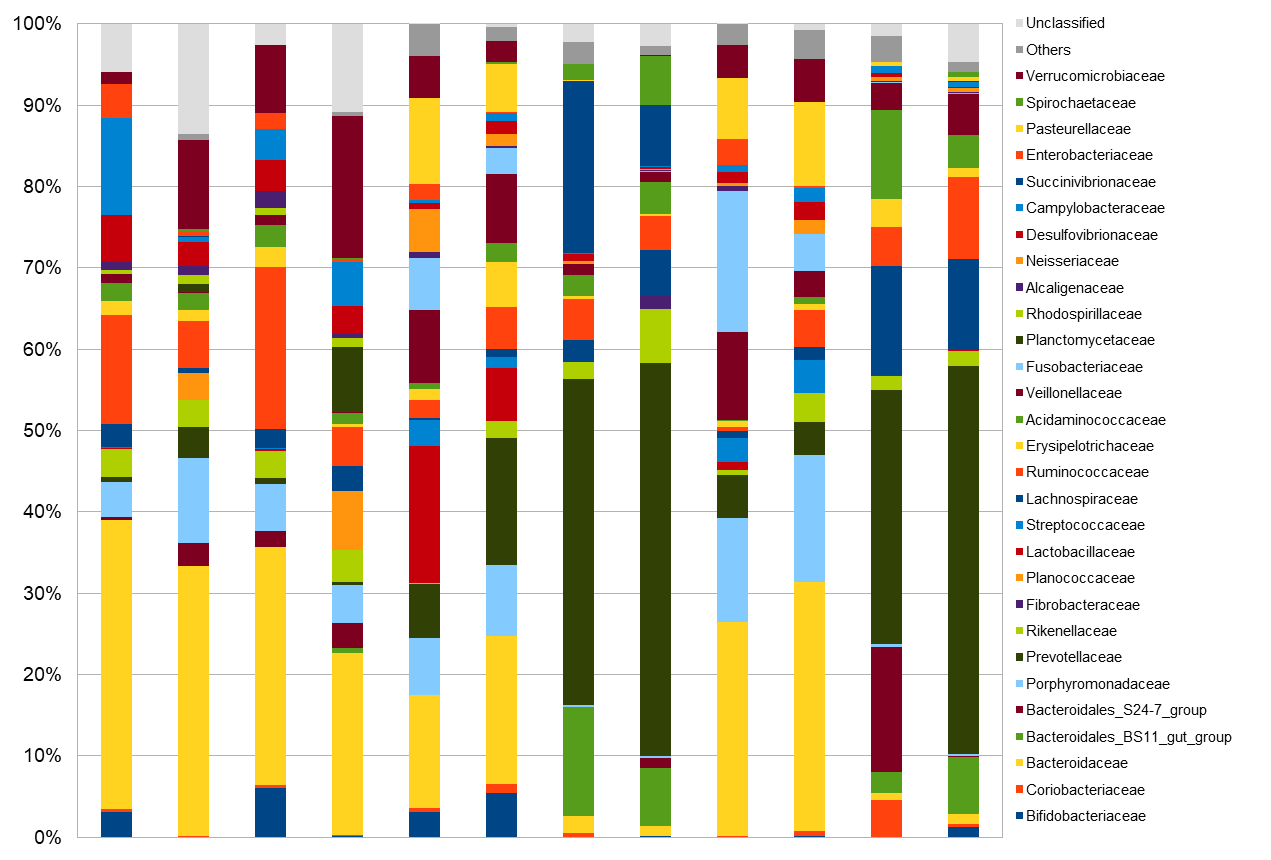


Control

Supplemented

Control

Supplemented

d35

d56

d35

d56

d7

d14

d35

d56

d7

d14

d35

d56

**Feces**

**Rumen**

Figure S4

**a)**


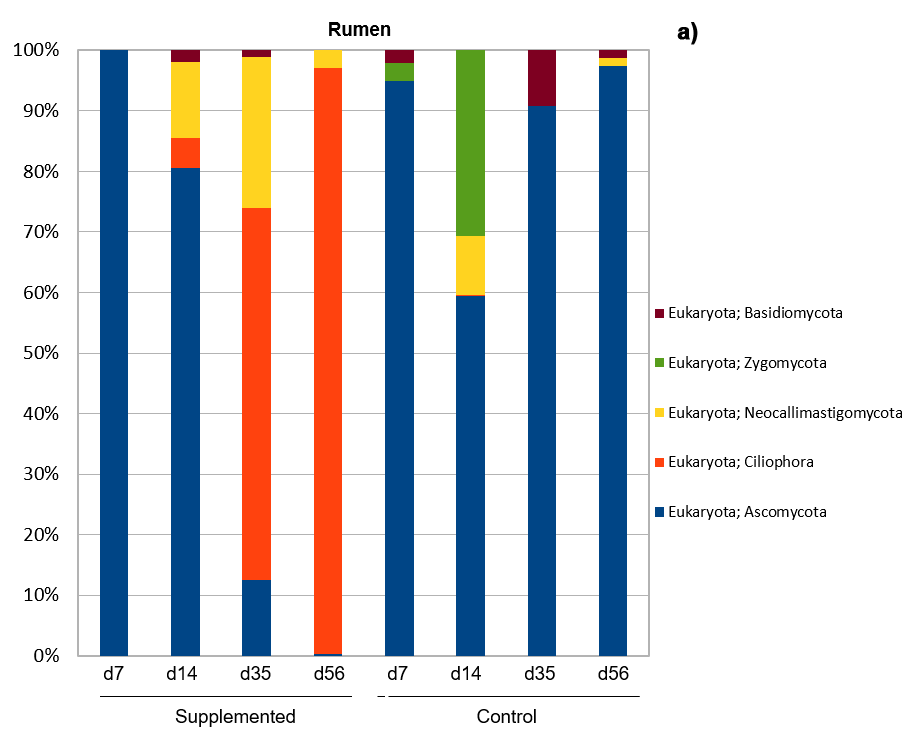

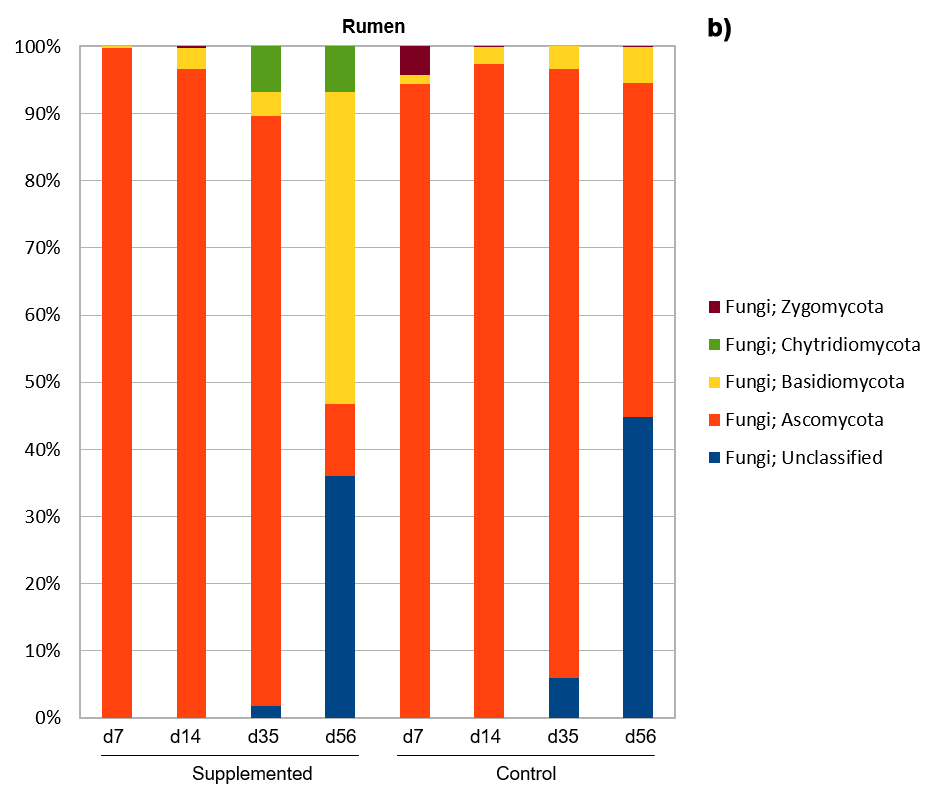


**b)**

**b)**

Figure S5

Figure S6

Birth

Weaning

d2 d3 d5 d7 d10 d14 d21 d28 d35 d42 d49 d56 d60

Colostrum

(d1)

Solid feed (starter concentrate)

Milk replacer (MR)

Rumen samples

Fecal samples

Figure S7

| **a)** |  | **Total bacteria** | | **Archaea** | | **Protozoa** | | | **Fungi** | |  |
| --- | --- | --- | --- | --- | --- | --- | --- | --- | --- | --- | --- |
|  | Age (days) | Control | Supplemented | Control | Supplemented | | Control | Supplemented | Control | Supplemented | |
|  | 2 | 8.61±0.18 | 8.22±1.02 | 5.05±2.48 | 5.78±0.65 | | nd | nd | nd | nd | |
|  | 3 | 8.71±0.51 | 8.56±0.57 | 5.96±0.24 | 6.27±0.72 | | nd | nd | nd | nd | |
|  | 5 | 7.40±0.61 | 8.03±0.79 | na | 3.65±3.36 | | nd | nd | nd | nd | |
|  | 7 | 8.83±0.33 | 8.45±0.60 | 6.25±0.32 | 4.49±3.82 | | nd | nd | nd | nd | |
|  | 10 | 8.89±0.28 | 8.39±0.55 | 5.38±1.41 | 4.28±2.40 | | nd | nd | nd | nd | |
|  | 14 | 8.56±0.10 | 8.46±0.16 | 4.61±2.40 | 5.17±2.69 | | nd | nd | nd | nd | |
|  | 21 | 8.56±0.17 | 8.41±0.83 | 6.57±0.54 | 6.79±0.82 | | nd | 1.95±3.22 | 1.92±2.18 | 3.83±2.16 | |
|  | 28 | 8.11±0.56 | 8.31±0.10 | 6.52±0.70 | 7.31±0.31 | | 1.08±1.76 | 3.83±2.76 | 1.86±2.05 | 2.80±1.78 | |
|  | 35 | 9.10±0.50 | 9.39±0.20 | 5.98±0.70 | 6.52±0.49 | | 2.73±2.25 | 6.36±1.90 | 0.87±1.35 | 2.39±2.13 | |
|  | 42 | 9.31±0.26 | 9.27±0.27 | 5.91±0.30 | 6.36±0.42 | | 1.84±2.02 | 5.32±3.01 | 0.73±1.78 | 1.96±1.59 | |
|  | 49 | 9.72±0.47 | 9.22±0.88 | 6.08±0.46 | 5.67±0.91 | | 2.55±2.10 | 6.67±1.45 | nd | 1.42±1.57 | |
|  | 56 | 9.55±0.46 | 9.81±0.32 | 6.01±0.29 | 6.29±0.63 | | 0.49±1.20 | 7.53±1.99 | nd | 1.57±2.45 | |

|  |  |  |  |  |  |  | |  |  |  | |  |
| --- | --- | --- | --- | --- | --- | --- | --- | --- | --- | --- | --- | --- |
| **b)** |  | ***Prevotella sp.*** | | ***Fibrobacter succinogenes*** | | ***Ruminococcus albus*** | | | ***Ruminococcus flavefaciens*** | |  |  |
|  | Age (days) | Control | Supplemented | Control | Supplemented | | Control | Supplemented | Control | Supplemented | | |
|  | 2 | 8.00±1.44 | 7.36±1.69 | 2.07±0.33 | 1.66±0.93 | | 1.50 ±1.41 | nd | 2.07 ±0.33 | 1.65±0.92 | | |
|  | 3 | 8.54±0.90 | 8.60±1.14 | 1.96±1.16 | 1.60±0.25 | | 0.74±0.93 | 0.39±0.48 | 2.11 ±1.72 | 1.45±1.63 | | |
|  | 5 | 7.28±1.38 | 8.26±0.98 | 1.64±1.54 | 1.89±0.51 | | nd | nd | 1.67 ±1.07 | 0.91±1.37 | | |
|  | 7 | 8.29±0.28 | 8.35±1.55 | 2.00±0.25 | 1.88±0.34 | | nd | 0.23±0.56 | 3.55 ±0.58 | 2.95±0.29 | | |
|  | 10 | 8.34±0.33 | 8.56±1.02 | 2.36±0.32 | 2.09±0.46 | | 1.46 ±1.62 | 2.73±0.60 | 3.51 ±0.76 | 4.79±2.17 | | |
|  | 14 | 8.14±1.20 | 8.05±0.40 | 2.02±0.32 | 2.38±0.34 | | 2.08±0.22 | 2.90±1.82 | 5.84 ±1.81 | 6.20±1.86 | | |
|  | 21 | 8.09±0.14 | 8.40±0.25 | 1.00±1.00 | 0.79±1.10 | | 5.11 ±1.64 | 5.33 ±1.93 | 7.72 ±1.73 | 7.64±0.98 | | |
|  | 28 | 8.14±1.72 | 8.36±0.11 | 1.22±1.34 | 2.21±0.40 | | 5.50 ±1.29 | 6.29 ±0.32 | 6.72 ±1.06 | 6.29±1.10 | | |
|  | 35 | 8.32±0.58 | 8.09±0.32 | 1.62±0.79 | 2.01±0.38 | | 5.37 ±1.53 | 5.59 ±1.11 | 7.26 ±1.01 | 5.84±1.48 | | |
|  | 42 | 8.67±0.20 | 8.65±0.31 | 2.19±0.69 | 4.16±2.54 | | 5.08 ±0.89 | 5.91 ±0.66 | 6.86 ±1.44 | 6.41±2.44 | | |
|  | 49 | 8.78±0.31 | 8.82±0.61 | 2.94±0.56 | 6.95±1.06 | | 6.24 ±0.81 | 6.23 ±1.10 | 7.74 ±0.96 | 7.57±1.44 | | |
|  | 56 | 8.48±0.44 | 9.06±0.42 | 2.55±0.81 | 7.66±0.70 | | 5.84 ±1.18 | 6.48 ±0.52 | 7.72 ±1.09 | 8.04±1.56 | | |

Table S1

| **Bacteria** | Control | | | |  | Supplemented | | | |
| --- | --- | --- | --- | --- | --- | --- | --- | --- | --- |
| Lamb age (days) | Observed OTUs | | Chao | Shannon |  | Observed OTUs | Chao | Shannon |  |
| 7 | 152 | | 185 | 3.87 |  | 140 | 171 | 3.77 |  |
| 14 | 185 | | 238 | 4.25 |  | 165 | 215 | 4.24 |  |
| 35 | 159 | | 192 | 4.33 |  | 167 | 192 | 4.11 |  |
| 56 | 195 | | 234 | 4.50 |  | 192 | 222 | 4.21 |  |
| Age effect | P<0.01 | | P<0.05 | ns |  |  |  |  |  |
| SC effect | ns | | ns | ns |  |  |  |  |  |
| Interaction | ns | | ns | ns |  |  |  |  |  |
|  |  | |  |  |  |  |  |  |  |
| **Eukaryota** | Control | | | |  | Supplemented | | | |
| Lamb age (days) | Observed OTUs | | Chao | Shannon |  | Observed OTUs | Chao | Shannon |  |
| 7 | 23 | | 33 | 1.63 |  | 45 | 64 | 2.17 |  |
| 14 | 17 | | 23 | 1.99 |  | 36 | 52 | 1.84 |  |
| 35 | 12 | | 13 | 1.20 |  | 15 | 19 | 1.31 |  |
| 56 | 7 | | 8 | 0.92 |  | 12 | 15 | 1.15 |  |
| Age effect | P<0.0001 | | P<0.0001 | P<0.05 |  |  |  |  |  |
| SC effect | P<0.001 | | P<0.001 | ns |  |  |  |  |  |
| Interaction | ns | | ns | ns |  |  |  |  |  |
|  |  | |  |  |  |  |  |  |  |
| **Fungi** | Control | | | |  | Supplemented | | | |
| Lamb age (days) | Observed OTUs | | Chao | Shannon |  | Observed OTUs | Chao | Shannon |  |
| 7 | 10 | | 11 | 1.90 |  | 17 | 20 | 2.03 |  |
| 14 | 10 | | 12 | 1.83 |  | 23 | 38 | 2.32 |  |
| 35 | 7 | | 7 | 1.28 |  | 10 | 10 | 1.66 |  |
| 56 | 6 | | 6 | 0.95 |  | 7 | 9 | 1.35 |  |
| Age effect | P<0.0001 | | P<0.0001 | P<0.05 |  |  |  |  |  |
| SC effect | P<0.001 | | P<0.001 | P<0.10 |  |  |  |  |  |
| Interaction | P<0.05 | | P<0.01 | ns |  |  |  |  |  |
|  |  |  | | |  |  |  |  |  |
| **Archaea** | Control | | | |  | Supplemented | | | |
| Lamb age (days) | Observed OTUs | | Chao | Shannon |  | Observed OTUs | Chao | Shannon |  |
| 7 | 0 | | 0 | 0.14 |  | 0 | 0 | 0 |  |
| 14 | 5 | | 10 | 0.37 |  | 14 | 39 | 0.38 |  |
| 35 | 31 | | 43 | 1.24 |  | 42 | 56 | 1.21 |  |
| 56 | 21 | | 29 | 0.73 |  | 26 | 35 | 0.87 |  |
| Age effect | P<0.0001 | | P<0.001 | P<0.0001 |  |  |  |  |  |
| SC effect | ns | | ns | ns |  |  |  |  |  |
| Interaction | ns | | ns | ns |  |  |  |  |  |

Table S2

| **Bacteria** | Control | | |  | | Supplemented | | | | |
| --- | --- | --- | --- | --- | --- | --- | --- | --- | --- | --- |
| Lamb age (days) | Observed OTUs | Chao | Shannon | |  | | Observed OTUs | Chao | Shannon |  |
| 35 | 211 | 270 | 4.59 | |  | | 192 | 237 | 4.36 |  |
| 56 | 210 | 269 | 4.71 | |  | | 228 | 265 | 4.87 |  |
|  |  |  |  | |  | |  |  |  |  |
| SC effect | ns | ns | ns | |  | |  |  |  |  |
|  |  |  |  | |  | |  |  |  |  |
|  |  |  |  | |  | |  |  |  |  |
| **Eukaryota** | Control | | |  | | Supplemented | | | | |
| Lamb age (days) | Observed OTUs | Chao | Shannon | |  | | Observed OTUs | Chao | Shannon |  |
| 35 | 12 | 16 | 0.95 | |  | | 25 | 33 | 1.64 |  |
| 56 | 10 | 15 | 0.87 | |  | | 17 | 20 | 1.42 |  |
|  |  |  |  | |  | |  |  |  |  |
| SC effect | P<0.01 | P<0.05 | P<0.01 | |  | |  |  |  |  |
|  |  |  |  | |  | |  |  |  |  |
|  |  |  |  | |  | |  |  |  |  |
| **Fungi** | Control | | |  | | Supplemented | | | | |
| Lamb age (days) | Observed OTUs | Chao | Shannon | |  | | Observed OTUs | Chao | Shannon |  |
| 35 | 15 | 22 | 1.17 | |  | | 11 | 16 | 1.21 |  |
| 56 | 2 | 3 | 0.07 | |  | | 10 | 18 | 1.25 |  |
|  |  |  |  | |  | |  |  |  |  |
| SC effect | ns | ns | ns (P=0.059) | |  | |  |  |  |  |
|  |  |  |  | |  | |  |  |  |  |
| **Archaea** | Control | | |  | | Supplemented | | | | |
| Lamb age (days) | Observed OTUs | Chao | Shannon | |  | | Observed OTUs | Chao | Shannon |  |
| 35 | 5 | 15 | 0.31 | |  | | 10 | 14 | 0.89 |  |
| 56 | 5 | 6 | 0.59 | |  | | 17 | 23 | 0.54 |  |
|  |  |  |  | |  | |  |  |  |  |
| SC effect | ns | ns | ns | |  | |  |  |  |  |
|  |  |  |  | |  | |  |  |  |  |

Table S3

| **Ingredient** | **%** |
| --- | --- |
| Barley | 21.5 |
| Soyabean meal | 18.0 |
| Corn | 16.0 |
| Wheat | 16.0 |
| Wheat bran | 10.0 |
| alfalfa | 6.5 |
| Beet pulp | 5.0 |
| Sugar cane molasses | 3.5 |
| Sodium Carbonate | 3.0 |
| Mineral supplement | 0.5 |
|  |  |
| **Chemical composition** | **g/kg of DM** |
| Starch | 389 |
| Sugars | 66 |
| NDF | 209 |
| ADF | 87 |
| ADL | 17 |
| CP | 188 |

Table S6

| **Microbial group/genus/species** | **Target gene** | **Primers** | **Reference** |
| --- | --- | --- | --- |
| Total bacteria | 16S rDNA | 5’-AGCAGCCGCGGTAAT-3’  5’-CAGGGTATCTAATCCTGTT-3’ | Bayat et al. |
| Fungi | ITS1 | 5’-GAGGAAGTAAAAGTCGTAACAAGGTTTC-3’  5’-CAAATTCACAAAGGGTAGGATGATT-3’ | Bayat et al. |
| Archaea | 16S rDNA | 5’-GAGGAAGGAGTGGACGACGGTA-3’  5’-ACGGGCGGTGTGTGCAAG-3’ | Bayat et al. |
| Protozoa | 18S rDNA | 5’-GCTTTCGWTGGTAGTGTATT-3’  5’-CTTGCCCTCYAATCGTWCT-3’ | Bayat et al. |
| *Prevotella sp.* | 16S rDNA | 5’-GGTTCTGAGAGGAAGGTCCCC-3’  5’-TCCTGCACGCTACTTGGCTG-3’ | Stevenson and Weimer 2007 |
| *Fibrobacter succinogenes* | 16S rDNA | 5’-GTTCGGAATTACTGGGCGTAAA-3’  5’-CGCCTGCCCCTGAACTATC-3’ | Bayat et al. |
| *Ruminococcus albus* | 16S rDNA | 5’-CCCTAAAAGCAGTCTTAGTTCG-3’  5’-CCTCCTTGCGGTTAGAACA-3’ | Mosoni et al. |
| *Ruminococcus flavefaciens* | 16S rDNA | 5’-CGAACGGAGATAATTTGAGTTTACTTAGG-3’  5’-CGGTCTCTGTATGTTATGAGGTATTACC-3’ | Bayat et al. |
| *Saccharomyces cerevisiae* | 26S rDNA | 5’- AGGAGTGCGGTTCTTTG -3’  5’- TACTTACCGAGGCAAGCTACA -3’ | Chang et al 2007 |

Table S7

References for Table S7:

Bayat, A. R. *et al.* Effect of camelina oil or live yeasts (*Saccharomyces cerevisiae*) on ruminal methane production, rumen fermentation, and milk fatty acid composition in lactating cows fed grass silage diets. *J. Dairy Sci.* **98**, 3166–3181 (2015).

Chang, H.W. *et al*. [Quantitative real time PCR assays for the enumeration of *Saccharomyces cerevisiae* and the *Saccharomyces sensu stricto* complex in human feces.](https://www.ncbi.nlm.nih.gov/pubmed/17900724) *J. Microbiol. Methods*. **71**, 191–201 (2007).

Chaucheyras-Durand, F. *et al.* Live yeasts enhance fibre degradation in the cow rumen through an increase in plant substrate colonization by fibrolytic bacteria and fungi. *J. Appl. Microbiol.* **120**, 560–570 (2016).

Mosoni, P., Martin, C., Forano, E. & Morgavi, D. P. Long-term defaunation increases the abundance of cellulolytic ruminococci and methanogens but does not affect the bacterial and methanogen diversity in the rumen of sheep. *J. Anim. Sci.* **89**, 783–791 (2011).

Stevenson, D.M. & Weimer, P.J. [Dominance of *Prevotella* and low abundance of classical ruminal bacterial species in the bovine rumen revealed by relative quantification real-time PCR.](https://www.ncbi.nlm.nih.gov/pubmed/17235560) *Appl. Microbiol. Biotechnol.* **75**, 165–174 (2007).

| **Microbial group** | **Targeted region** | **Primers** |
| --- | --- | --- |
| Bacteria | V4 region of the 16S rDNA | 515f-806r |
| Archaea | Region of the 16S rDNA | 349f-806r |
| Eukaryota | Region of the 18S rDNA | 566f-1200r |
| Fungi | ITS3-ITS4 | ITS3-ITS4 |

Table S8
